# Objective tongue phenotyping and nutritional risk in diabetic kidney disease: a dual-centre study linking quantified tongue features to the controlling nutritional status score

**DOI:** 10.3389/fnut.2026.1902694

**Published:** 2026-08-14

**Authors:** Zhaoxi Dong, Jiayou Liu, Jiyuan Hu, Jiaming Su, Zheyu Xu, Fengyi Cai, Weihong Chen, Qingqing Liu, Chengdong Peng, Yang Shi, Hongfang Liu

**Affiliations:** 1 Dongzhimen Hospital, Beijing University of Chinese Medicine, Beijing, China; 2 Beijing Hospital of Integrated Traditional Chinese and Western Medicine, Beijing, China; 3 School of Computer Science and Information Engineering, Hefei University of Technology, Hefei, Anhui, China; 4 Artificial Intelligence Laboratory, Hefei Yunzhen Information Technology Co., Ltd., Hefei, Anhui, China; 5 Treatment Center of Kidney Disease, The First Affiliated Hospital of Henan University of Chinese Medicine, Zhengzhou, China

**Keywords:** artificial intelligence, controlling nutritional status score, diabetic kidney disease, nutritional assessment, objective tongue diagnosis, protein-energy wasting, tongue features, traditional Chinese medicine

## Abstract

**Objective:**

To examine associations between objectively quantified tongue features and the Controlling Nutritional Status (CONUT) score in patients with diabetic kidney disease (DKD), and to assess the influence of renal function and glycaemic status on these associations.

**Methods:**

This dual-centre cross-sectional study included 392 patients with DKD. Fifty-one tongue features were extracted using the YZAI-02 AI tongue imaging system. CONUT was calculated from serum albumin, lymphocyte count, and total cholesterol after multiple imputation by chained equations (*m* = 20). Rubin-pooled partial Spearman correlations, adjusted single-feature and joint multivariable linear regression, sensitivity analyses, and ordinal logistic regression were performed. Covariates were selected a priori and included age, sex, study centre, estimated glomerular filtration rate (eGFR), and HbA1c. Exploratory stratified and interaction analyses were conducted according to eGFR and sex.

**Results:**

The median CONUT score was 3 (interquartile range: 2–5), and 304 patients (77.6%) had CONUT-defined malnutrition. Five of 51 tongue features reached nominal significance in partial-correlation screening, although none survived false discovery rate correction across all 51 comparisons. After collinearity pruning, five representative features met the exploratory false discovery rate threshold in adjusted single-feature models. In the joint multivariable model, higher edge tongue saturation (*β* = 0.269 per standard deviation, 95% CI: −0.023 to 0.560, *p* = 0.071) and lower middle tongue brightness (β = −0.234, 95% CI: −0.495 to 0.026, *p* = 0.078) showed borderline associations with higher CONUT scores. Adding eGFR increased the coefficient for edge tongue saturation from 0.131 to 0.289, consistent with statistical suppression, whereas the coefficient for middle tongue brightness remained stable. Additional proteinuria adjustment produced coefficient changes below 15%, and both estimates remained imprecise. In ordinal logistic regression, edge tongue saturation showed a directionally consistent borderline association (OR = 1.29, 95% CI: 0.96–1.71, *p* = 0.086). No interaction remained significant after false discovery rate correction.

**Conclusion:**

CONUT-defined malnutrition was common in DKD. Middle tongue brightness and edge tongue saturation showed modest, borderline associations with nutritional risk, suggesting that AI-assisted tongue phenotyping warrants further evaluation as a complement to nutritional surveillance in DKD.

## 1 Introduction

Diabetic kidney disease (DKD) is the leading cause of end-stage renal disease worldwide, affecting approximately 20–40% of patients with type 2 diabetes mellitus and accounting for a disproportionate share of the global burden of chronic kidney disease (CKD) (1, 2). Beyond its haemodynamic and structural renal consequences, DKD is characterised by a progressive and multifaceted metabolic deterioration that encompasses impaired protein and lipid metabolism, chronic low-grade inflammation, and dysregulation of immune homeostasis—processes that collectively predispose patients to protein-energy wasting (PEW) and clinically significant malnutrition (3, 4). Nutritional depletion in DKD is not merely a downstream consequence of renal failure; it is an active contributor to accelerated eGFR decline, increased susceptibility to infection, impaired wound healing, higher hospitalisation rates, and excess cardiovascular mortality (5, 6). Despite this substantial clinical burden, nutritional risk in DKD remains systematically underrecognised, partly because its manifestations overlap with the broader metabolic consequences of renal failure, and partly because malnutrition may be perceived as an inevitable rather than modifiable feature of advanced disease.

While the full diagnostic construct of PEW requires assessment across multiple domains including body composition and dietary intake, routine clinical practice frequently relies on biochemical surrogates to identify patients at nutritional risk. The Controlling Nutritional Status (CONUT) score, derived from serum albumin, peripheral blood lymphocyte count, and total cholesterol, was originally developed and validated as a simple, automated bedside tool for detecting hospital malnutrition that uses data available from routine biochemistry panels (7). Its three components collectively reflect the major axes of nutritional impairment: albumin captures protein synthesis capacity and visceral protein reserves; lymphocyte count indexes cellular immune competence and reflects protein-energy adequacy; and total cholesterol provides a surrogate measure of lipid-energy reserves and hepatic synthetic function. In the context of DKD, all three CONUT components are directly shaped by the renal-metabolic milieu: uraemic inflammation suppresses hepatic albumin synthesis, lymphopenia is a recognised consequence of advanced CKD and uraemic immunosuppression, and hypocholesterolaemia accompanies progressive protein-energy wasting as hepatic lipoprotein synthesis declines alongside nutritional depletion. These mechanistic links make CONUT clinically relevant in DKD, but also complicate its interpretation because higher scores may reflect renal disease severity and urinary protein loss in addition to nutritional depletion. Several recent studies have substantiated its prognostic value in DKD: higher CONUT scores have been independently associated with renal disease progression, cardiovascular events, and all-cause mortality in patients with biopsy-confirmed DKD (6), and with all-cause and cause-specific mortality in a nationally representative US cohort of DKD patients (8). Corroborating these findings, nutritional status assessed by multiple scoring instruments—including CONUT—has consistently predicted adverse outcomes in DKD (9). Importantly, a recent retrospective cohort study identified the CONUT score as an independent risk factor for progression from DKD to end-stage renal disease in patients with type 2 diabetes mellitus (10), underscoring its relevance not only for risk stratification but also as a potential target for intervention to slow disease progression. Nevertheless, the CONUT score still requires venepuncture and laboratory analysis, which may not be available at every patient contact, particularly in resource-limited settings or during community-based follow-up visits.

Traditional Chinese medicine (TCM) tongue diagnosis is a millennia-old clinical inspection technique in which the colour, texture, moisture, and coating characteristics of the tongue body are systematically evaluated as a non-invasive window into the functional status of internal organs and the balance of qi, blood, yin, and yang. In TCM theory, the tongue is uniquely accessible as a vascularised mucosal surface that reflects systemic physiological and pathological states: the tongue body colour and brightness are held to mirror the state of qi and blood, the tongue coating reflects the function of the spleen and stomach in transforming and transporting food essence, and different tongue regions are attributed to different organ systems—the centre to the spleen and stomach, the edges to the liver and gallbladder, the tip to the heart and lungs, and the root to the kidney (11). The plausibility of these anatomical correspondences is increasingly supported by objective studies demonstrating that quantitative tongue colour parameters correlate with haematological, inflammatory, and metabolic biomarkers in chronic disease populations (12–15).

The advent of AI-assisted tongue imaging systems has transformed tongue diagnosis from a subjective, observer-dependent skill into a reproducible, quantitative assessment capable of generating dozens of colorimetric and textural parameters from a single tongue photograph (16, 17). This technological advancement has enabled rigorous cross-sectional and longitudinal research linking tongue phenotypes to clinical biomarkers and disease outcomes. Recent work has extended this approach to nutritional medicine: objective tongue features extracted by AI have been associated with gut microbiome changes during dietary intervention (18) and have predicted nutritional risk in cancer patients undergoing radiochemotherapy with high accuracy (19), suggesting that quantitative tongue imaging may serve as a non-invasive nutritional surveillance tool across a range of clinical contexts. In DKD specifically, prior work from our group has demonstrated that objective tongue features are associated with the atherogenic index of plasma—a marker of dyslipidaemia and cardiovascular risk—with findings indicating that tongue centre and tip colour saturation and brightness are sensitive to metabolic deterioration in this population (20). We have further shown that tongue features differ systematically across CKD stages within DKD patients classified by damp-heat syndrome, with progressive changes in tongue edge pallor and coating brightness accompanying advancing renal insufficiency (21). These studies establish the biological plausibility of tongue–biomarker associations in DKD, but have not examined whether tongue features reflect nutritional status as quantified by a validated scoring system.

The intersection of TCM tongue diagnosis and nutritional medicine in DKD represents a clinically meaningful but largely unexplored research space. If objective tongue features are systematically associated with CONUT-defined nutritional risk, they could serve as a non-invasive, real-time complement to laboratory-based nutritional screening—enabling clinicians to identify patients at nutritional risk between blood tests, to monitor nutritional trajectories dynamically, and to enrich TCM syndrome differentiation with objective nutritional biomarker data. Furthermore, understanding which tongue features reflect nutritional status and whether their associations are independent of renal function would clarify the mechanistic pathways linking tongue appearance to systemic health in DKD, advancing the theoretical basis of objective tongue phenotyping as a complementary assessment tool in the integrative management of DKD.

To address this gap, we conducted a dual-centre cross-sectional study enrolling 392 patients with DKD from two tertiary referral centres in Beijing. Using the YZAI-02 AI tongue imaging system, we extracted 51 objective tongue features and examined their associations with CONUT score across multiple analytical frameworks: Rubin-pooled partial Spearman correlations, univariate and multivariable linear regression, sensitivity analyses including proteinuria adjustment and nested model analysis, and Rubin-pooled ordinal logistic regression. Our primary aims were: (1) to characterise the distribution and clinical correlates of CONUT-defined nutritional risk; (2) to evaluate associations between objective tongue features and continuous CONUT scores after adjustment for demographic characteristics, study centre, renal function, and glycaemic status; (3) to assess how eGFR, HbA1c, and proteinuria influenced the estimated tongue–CONUT associations; and (4) to explore potential effect modification according to renal function and sex. Given the cross-sectional design and multiple feature comparisons, all tongue-feature findings were considered exploratory.

## 2 Methods

### 2.1 Study design and participants

This cross-sectional study consecutively enrolled inpatients and outpatients with clinically confirmed DKD from two tertiary referral centres in Beijing, China: Beijing University of Chinese Medicine Dongzhimen Hospital and Beijing Hospital of Integrated Traditional Chinese and Western Medicine, between May 2023 and May 2026. Of the 392 enrolled participants, 338 (86.2%) were recruited from Dongzhimen Hospital (centre 1) and 54 (13.8%) from Beijing Hospital of Integrated Traditional Chinese and Western Medicine (centre 2). The study protocol was approved by the Ethics Committee of Dongzhimen Hospital, Beijing University of Chinese Medicine (approval number: 2021DZMEC-209-08), and all participants provided written informed consent prior to enrolment.

DKD was diagnosed according to the Chinese Guidelines for the Clinical Diagnosis and Treatment of Diabetic Kidney Disease (2021 edition) and the chronic kidney disease framework defined in the Kidney Disease: Improving Global Outcomes (KDIGO) 2022 Clinical Practice Guideline for Diabetes Management in CKD, defined as persistent structural or functional kidney abnormalities for more than 3 months, manifested as sustained albuminuria and/or reduced estimated glomerular filtration rate (eGFR) (22). In clinical practice, DKD was identified based on a history of diabetes mellitus, albuminuria or proteinuria, eGFR, and serum creatinine, with careful exclusion of other clearly defined non-diabetic kidney diseases.

Inclusion criteria were: (1) age 18–90 years, irrespective of sex; (2) fulfilment of the DKD diagnostic criteria; (3) ability to cooperate with tongue image acquisition; and (4) provision of written informed consent. Exclusion criteria were: (1) type 1 diabetes mellitus; (2) clearly defined non-diabetic kidney disease, including primary glomerulonephritis, latent nephritis, autoimmune diseases and connective tissue disorders, malignancies, or drug-induced secondary renal injury; (3) recent critical illness within the preceding 6 months, including malignant hypertension, myocardial infarction, or cerebrovascular accident; (4) severe primary diseases of the respiratory, digestive, or haematopoietic systems, serious infection, or psychiatric disorders; (5) pregnancy or lactation; and (6) obvious tongue discolouration attributable to medications, food, or other extrinsic factors that could confound tongue image interpretation.

### 2.2 Tongue image acquisition and feature extraction

Tongue images were acquired using the YZAI-02 Chinese medicine AI tongue imaging system, which incorporates a standard LED ring light, a portable acquisition unit with a multi-curved face-fitting design to prevent ambient light interference, an ultra-high-resolution camera, and an Android-based host unit. Prior to data collection, all operators received standardised training to ensure procedural consistency and minimise operator-dependent error. Participants were seated upright, facing the device, with their chin resting against the lower edge of the acquisition port. They were instructed to open their mouth naturally, protrude the tongue without muscular tension, lower the tongue tip slightly, and flatten the tongue surface to fully expose the tongue body for 2–3 s before image capture was triggered by the attending physician. If image quality was suboptimal or tongue exposure was insufficient, a brief rest period was allowed before reacquisition.

Each image was synchronously uploaded to the AI Open Platform for Tongue Diagnosis in Traditional Chinese Medicine (Chinese invention patent nos. CN202211523344.1, CN202211315242.0, and CN201910568779. X) for automated analysis. Following colour correction, the platform performed tongue body segmentation and separation of the tongue coating from the tongue body, followed by multidimensional feature extraction. Colour features were quantified in the hue-saturation-value (HSV) colour model (H: 0–180; S: 0–255; V: 0–255) and the red-green-blue (RGB) colour model (0–255). Tongue colour and coating colour features were extracted from four anatomical regions: tongue tip, tongue centre, tongue root, and tongue sides, with mean H, S, and V values computed for each region together with global tongue-level HSV values. Tongue body features included the presence or absence of tooth marks and fissures. Tongue coating features included coating ratio, coating thickness grade (none/mild/moderate/severe, re-coded as 0/1/2/3), and coating texture features derived from first-, second-, and third-order colour moments (in the RGB colour space) and grey-level co-occurrence matrix (GLCM) descriptors, including correlation (COR), contrast (CON), energy, angular second moment (ASM), inverse difference moment (IDM), entropy, roughness, and grey mean. The final dataset comprised 48 continuous tongue features (coating_ratio and 47 HSV/texture parameters), one ordinal variable (coating thickness grade), and two binary variables (tooth marks and fissures), yielding 51 tongue variables in total.

### 2.3 Clinical data collection

Demographic information (sex and age) and contemporaneous laboratory test results were extracted from the electronic medical record system for each participant. Laboratory measurements included complete blood counts, coagulation parameters, urinalysis, liver function parameters, kidney function parameters, electrolytes, lipid profile, glycaemic parameters, inflammatory markers, 24-h urinary protein quantification, and urinary albumin-to-creatinine ratio (UACR). Values reported as below the assay detection limit (e.g., CRP < 1 mg/L or <0.2 mg/L) were treated as left-censored and replaced with half the respective detection limit prior to missingness assessment. The eGFR was estimated using the CKD-EPI equation incorporating serum creatinine (23).

### 2.4 CONUT score calculation

The CONUT score was calculated for each participant according to the original validated algorithm (7). Three laboratory components were used: serum albumin (ALB, g/L), peripheral blood lymphocyte count (LY#, ×10^9^/L), and total cholesterol (TC, mmol/L). Subscores were assigned as follows: albumin subscore—0 points for ALB ≥35 g/L, 2 points for 30–34.9 g/L, 4 points for 25–29.9 g/L, and 6 points for <25 g/L; lymphocyte subscore—0 points for LY# ≥ 1.6 × 10^9^/L, 1 point for 1.2–1.59 × 10^9^/L, 2 points for 0.8–1.19 × 10^9^/L, and 3 points for <0.8 × 10^9^/L; total cholesterol subscore—0 points for TC ≥ 5.18 mmol/L, 1 point for 4.14–5.17 mmol/L, 2 points for 3.11–4.13 mmol/L, and 3 points for <3.11 mmol/L. The total CONUT score ranges from 0 to 12, with higher scores indicating worse nutritional status. Patients were classified into four nutritional categories: normal (0–1), light malnutrition (2–4), moderate malnutrition (5–8), and severe malnutrition (9–12). For primary analyses, patients were also dichotomised into normal nutritional status (CONUT 0–1) and any-degree malnutrition (CONUT ≥2). CONUT was treated as a continuous outcome variable (range 0–12) in all regression analyses.

### 2.5 Missing data handling

Missing laboratory data were handled using the multiple imputation by chained equations (MICE) framework. Laboratory variables with missingness exceeding 30% were excluded from all analyses; in this cohort, erythrocyte sedimentation rate (ESR, 65.8% missing), homocysteine (HCY, 60.2% missing), urinary albumin-to-creatinine ratio (UACR, 45.9% missing), cystatin C (CysC, 34.9% missing), and prealbumin (PA, 32.9% missing) were excluded on this basis. Of the three CONUT component variables, albumin had 3.6% missing, lymphocyte count had 3.1% missing, and total cholesterol had 11.5% missing; all were well below the exclusion threshold and were therefore included in the imputation framework. For all retained laboratory variables with missingness ≤30%, 20 imputed datasets were generated using predictive mean matching for continuous variables (m = 20, maxit = 10), with sex, centre, and all available clinical variables included as predictors (24). The CONUT score and its categorical classifications were computed within each imputed dataset after imputation. Effect estimates from all 20 imputed datasets were pooled using Rubin’s rules to yield combined point estimates, standard errors, 95% confidence intervals, and *p*-values (25). The stability of CONUT estimates across imputations was verified by inspecting the between-imputation range of mean and median CONUT values. All 51 tongue variables were complete without missing values and required no imputation. Although 24-h urinary protein (U24h_protein; 20.9% missing) was retained and imputed, it was not incorporated as a primary covariate alongside eGFR for three reasons: first, the absence of UACR precluded simultaneous adjustment for both established indices of glomerular proteinuria in the same model; second, current international guidelines preferentially recommend UACR over 24-h urinary protein for routine CKD monitoring and risk stratification given its superior standardisation and practicability; and third, given that both eGFR and U24h_protein reflect overlapping dimensions of glomerular injury, their simultaneous inclusion as primary covariates risks over-adjustment and suppression of the nutritional signal of interest. U24h_protein was therefore retained exclusively as an additional covariate in a pre-specified sensitivity analysis.

### 2.6 Statistical analysis

Continuous variables are presented as median (interquartile range [IQR]) and categorical variables as frequency (percentage). Between-group comparisons of continuous variables were conducted using the Wilcoxon rank-sum test for two-group comparisons and the Kruskal–Wallis test for comparisons across four nutritional categories; categorical variables were compared using the Pearson chi-square test, as appropriate.

Covariates were selected *a priori* on the basis of clinical relevance and their potential relationships with both tongue appearance and CONUT. Age and sex were included as fundamental demographic factors; study centre was included to account for differences in recruitment setting and centre-specific case mix; eGFR was included because renal disease severity may influence both tongue appearance and the laboratory components of CONUT; and HbA1c was included to account for glycaemic status. Covariate selection was not based on statistical significance in the current dataset. Because CONUT components may also be affected by urinary protein loss, proteinuria was evaluated through an additional sensitivity model.

To assess the associations between all 51 tongue features and continuous CONUT score, Rubin-pooled partial Spearman correlations were computed across 20 imputed datasets. Partial correlations were estimated via a rank-based residualisation approach: within each imputed dataset, both the tongue feature and CONUT were converted to ranks, and each rank vector was then regressed on a set of pre-specified covariates—chronological age, sex, study centre, eGFR, and HbA1c—using ordinary least squares. Pearson correlation of the resulting residuals yielded the partial Spearman correlation coefficient. Fisher z-transformation was applied to each estimate, and the transformed values were pooled using Rubin’s rules with variance approximation 1/(n − k − 3), where k denotes the number of covariates. This approach was applied uniformly to all tongue variable types (continuous, ordinal, and binary) to ensure analytical consistency. This approach yields a partial Spearman correlation that is robust to non-normality and ordinal outcomes. The Benjamini–Hochberg (BH) false discovery rate (FDR) procedure was applied to correct for multiple comparisons across all 51 tongue features (26). Tongue features with an unadjusted pooled *p*-value < 0.10 were retained as candidates for subsequent regression analysis.

Before multivariable modelling, candidate features were subjected to collinearity pruning using hierarchical clustering with complete linkage applied to the absolute pairwise Pearson correlation matrix computed from the first imputed dataset. Features were merged into clusters at a threshold of |r| ≥ 0.80, and the representative variable from each cluster (i.e., the feature with the lowest unadjusted *p*-value within the cluster) was selected. Candidate tongue features were z-score standardised using the mean and standard deviation of the first imputed dataset as reference values, so that regression coefficients reflect the change in CONUT score per one standard deviation increment in the tongue feature. Rubin-pooled single-feature linear regression models were then fitted separately for each cluster representative, with adjustment for age, sex, centre, eGFR, and HbA1c. Features reaching BH FDR-adjusted *p* < 0.10 in univariate regression—and at most five features to avoid overfitting—were entered simultaneously into a pooled multivariable linear regression model with the same covariate set. Variance inflation factors (VIFs) were calculated from the first imputed dataset to assess residual multicollinearity in the multivariable model.

Three pre-specified sensitivity analyses were conducted. First, the multivariable model was re-fitted after removing eGFR and HbA1c from the covariate set, retaining only age, sex, and centre, to evaluate whether tongue–CONUT associations were robust to exclusion of renal and glycaemic adjustment variables. Second, given that U24h_protein was not included as a primary covariate for the reasons described above, log-transformed 24-h urinary protein (log[1 + U24h_protein]) was added to the full covariate set as a sensitivity check to assess whether the tongue–CONUT associations persisted after additional proteinuria adjustment; the percentage change in each tongue feature coefficient relative to the primary model was used to quantify the influence of proteinuria. Third, nested model analysis was performed by progressively adding eGFR (Model 1: age + sex + centre) and then HbA1c (Model 2: age + sex + centre + eGFR; Model 3: age + sex + centre + eGFR + HbA1c) to characterise whether eGFR and HbA1c acted as confounders (attenuating tongue feature coefficients upon adjustment) or suppressors (amplifying tongue feature coefficients upon adjustment) (27).

To complement the primary continuous outcome analysis, Rubin-pooled ordinal logistic regression was performed using the proportional odds model (R package MASS:polr), with CONUT nutritional category (normal / light / moderate / severe, treated as an ordered outcome) as the dependent variable and the same five candidate tongue features and covariates as the primary multivariable model. Coefficients were estimated on the log-odds scale within each imputed dataset and pooled across 20 datasets using Rubin’s rules; pooled log-odds were exponentiated to yield odds ratios (ORs) with 95% confidence intervals.

Exploratory effect-modification analyses were conducted according to renal function and sex. For renal function, joint models were fitted separately for patients with eGFR ≥45 and <45 mL/min/1.73 m^2^, and formal interaction models treated eGFR as a continuous variable centred at 45 and scaled per 10 mL/min/1.73 m^2^. Sex-stratified joint models and tongue feature-by-sex interaction models were also fitted. All five tongue features were entered simultaneously in these models. Estimates were pooled across 20 imputed datasets using Rubin’s rules with Barnard–Rubin small-sample degrees of freedom. BH correction was applied separately across the five interaction tests within each modifier family. Differences in subgroup-specific *p*-values were not interpreted as evidence of heterogeneity without a corresponding interaction effect.

The sample size was determined by the number of eligible participants enrolled during the study period; no formal *a priori* sample-size calculation was performed. Precision was assessed using effect estimates and 95% confidence intervals, and no *post hoc* power calculation was performed. Because the severe CONUT category included only 11 participants and CONUT category was the study outcome, nutritional-category subgroup regressions were not performed; category-based results were limited to descriptive analyses.

All analyses were performed in R version 4.4.1 (R Foundation for Statistical Computing, Vienna, Austria), using the packages mice, MASS, dplyr, ggplot2, gtsummary, and gt. All statistical tests were two-tailed; raw *p* < 0.05 was considered nominally significant, and BH-adjusted p < 0.05 was considered significant after multiple-comparison correction. The thresholds of raw *p* < 0.10 during screening and BH-adjusted p < 0.10 during adjusted single-feature modelling were used only as exploratory feature-selection criteria and were not interpreted as confirmatory statistical significance.

## 3 Results

### 3.1 Participant characteristics and CONUT score distribution

**Figure 1:**
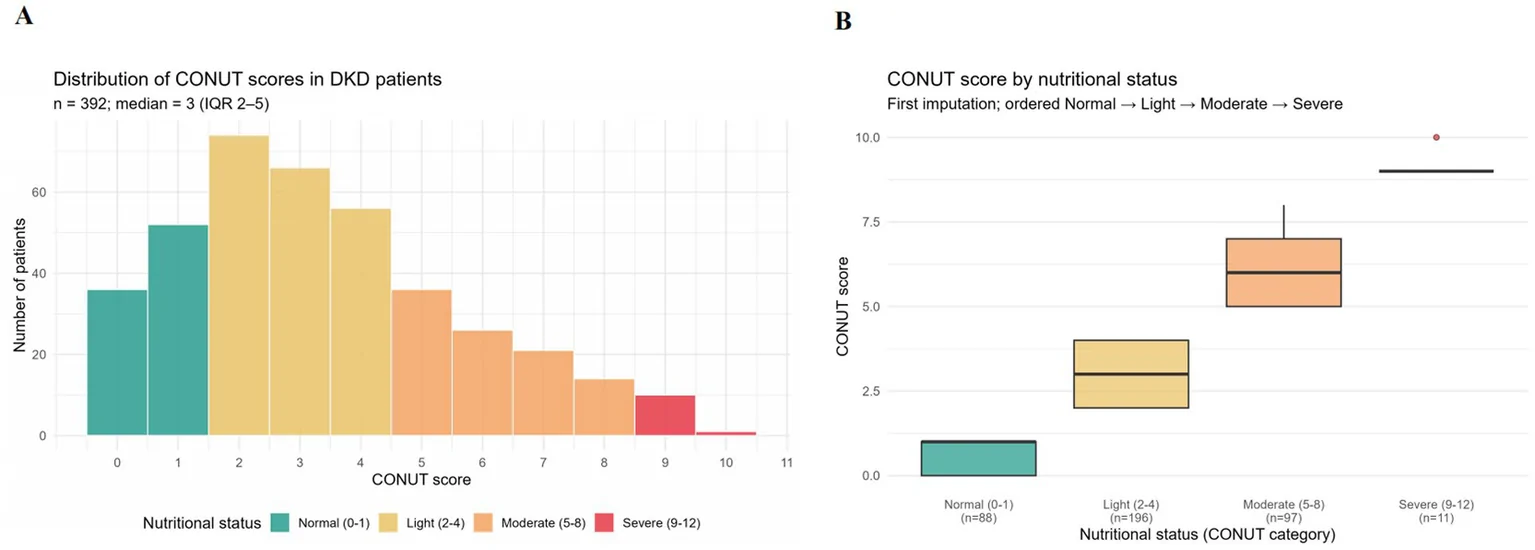
Distribution of CONUT scores in patients with diabetic kidney disease. **(A)** Histogram of CONUT scores (first imputed dataset; *n* = 392). Bars are colour-coded by nutritional category: Normal (green, 0–1), Light malnutrition (yellow, 2–4), Moderate malnutrition (orange, 5–8), and Severe malnutrition (red, 9–12). **(B)** Box plots of CONUT scores stratified by nutritional category, presented in ascending order of severity. Boxes represent the interquartile range (IQR), horizontal lines indicate the median, and whiskers extend to 1.5 × IQR; individual points beyond the whiskers are shown as outliers. Sample sizes per category are indicated below each box label. CONUT, Controlling Nutritional Status score; IQR, interquartile range.

A total of 392 patients with DKD were included in this analysis (centre 1, *n* = 338; centre 2, *n* = 54). The median age was 62 years (IQR 54–70), and 269 participants (68.6%) were male. The median CONUT score was 3 (IQR 2–5). Overall, 304 patients (77.6%) exhibited some degree of malnutrition (CONUT ≥ 2), comprising 196 (50.0%) with light malnutrition (CONUT 2–4), 97 (24.7%) with moderate malnutrition (CONUT 5–8), and 11 (2.8%) with severe malnutrition (CONUT 9–12); 88 patients (22.4%) had normal nutritional status (CONUT 0–1). The distribution of CONUT scores was right-skewed, with the modal category falling in the light malnutrition range (Figures 1A,B).

Baseline clinical characteristics stratified by nutritional status (normal vs. malnutrition) are presented in Table 1. Compared with patients in the normal group, those with malnutrition were significantly older (median 62 vs. 58 years, *p* < 0.001) and had markedly worse renal function, with a lower eGFR (median 34 vs. 71 mL/min/1.73 m^2^, *p* < 0.001), higher serum creatinine (172 vs. 98 μmol/L, *p* < 0.001), and higher blood urea nitrogen (12 vs. 9 mmol/L, *p* < 0.001). Nutritional and haematological parameters were substantially impaired in the malnutrition group, including lower albumin (35.5 vs. 41.3 g/L, *p* < 0.001), lower lymphocyte count (1.34 vs. 1.96 × 10^9^/L, *p* < 0.001), lower total cholesterol (3.86 vs. 5.44 mmol/L, *p* < 0.001), lower haemoglobin (112 vs. 133 g/L, *p* < 0.001), and lower calcium (2.20 vs. 2.34 mmol/L, p < 0.001). Proteinuria was more severe in the malnutrition group, as reflected by higher 24-h urinary protein quantification (2,685 vs. 641 mg/24 h, *p* < 0.001) and a higher proportion of patients with high-grade urine protein on dipstick (*p* = 0.002). Triglycerides (1.67 vs. 2.22 mmol/L, *p* < 0.001), LDL-C (2.21 vs. 3.15 mmol/L, *p* < 0.001), and CO₂CP (*p* = 0.004) also differed significantly between groups.

**Table 1:** Baseline clinical characteristics stratified by nutritional status.

Variable	Overall (*n* = 392)	Normal (*n* = 88)	Malnutrition (*n* = 304)	*p* value	Sig
Sex	Male: 269 (68.6%); female: 123 (31.4%)	Male: 53 (60.2%); female: 35 (39.8%)	Male: 216 (71.1%); female: 88 (28.9%)	0.054	
Age (years)	62.0 (54.0–70.0)	58.0 (46.8–68.0)	62.0 (56.0–71.0)	<0.001	***
Study centre	center1: 338 (86.2%); center2: 54 (13.8%)	center1: 80 (90.9%); center2: 8 (9.1%)	center1: 258 (84.9%); center2: 46 (15.1%)	0.148	
eGFR (mL/min/1.73 m^2^)	41.4 (15.6–73.4)	70.7 (40.5–96.0)	33.5 (12.9–60.8)	<0.001	***
Serum creatinine (μmol/L)	151.1 (90.2–355.1)	97.7 (76.5–157.9)	172.4 (102.0–406.0)	<0.001	***
Blood urea nitrogen (mmol/L)	11.5 (7.6–18.7)	9.0 (6.6–13.3)	12.4 (8.5–20.1)	<0.001	***
Serum albumin (g/L)	37.4 (32.7–41.5)	41.2 (37.9–43.8)	35.5 (31.4–40.1)	<0.001	***
Lymphocyte count (×10^9^/L)	1.5 (1.1–1.9)	2.0 (1.7–2.2)	1.3 (1.0–1.7)	<0.001	***
Total cholesterol (mmol/L)	4.3 (3.4–5.4)	5.4 (4.9–6.1)	3.9 (3.2–5.0)	<0.001	***
Haemoglobin (g/L)	117.0 (101.0–135.0)	133.0 (122.0–149.2)	112.0 (97.0–129.0)	<0.001	***
Triglycerides (mmol/L)	1.8 (1.2–2.6)	2.2 (1.4–3.1)	1.7 (1.1–2.4)	<0.001	***
LDL-C (mmol/L)	2.4 (1.9–3.1)	3.1 (2.7–3.8)	2.2 (1.7–2.8)	<0.001	***
HDL-C (mmol/L)	1.1 (0.9–1.3)	1.1 (1.0–1.3)	1.0 (0.9–1.3)	0.039	*
Calcium (mmol/L)	2.2 (2.1–2.4)	2.3 (2.3–2.4)	2.2 (2.1–2.3)	<0.001	***
Phosphorus (mmol/L)	1.3 (1.2–1.5)	1.3 (1.1–1.4)	1.3 (1.2–1.5)	0.023	*
Potassium (mmol/L)	4.4 (4.0–4.8)	4.3 (3.9–4.5)	4.4 (4.0–4.8)	0.013	*
CO₂CP (mmol/L)	24.7 (22.5–27.0)	25.5 (23.9–27.3)	24.4 (22.1–27.0)	0.004	**
Fasting glucose (mmol/L)	7.4 (5.9–9.9)	8.6 (6.4–11.0)	7.1 (5.8–9.6)	0.006	**
HbA1c (%)	7.3 (6.4–8.6)	8.5 (7.3–9.8)	7.1 (6.3–8.1)	<0.001	***
CRP (mg/L)	0.8 (0.5–4.1)	0.5 (0.5–2.9)	1.0 (0.5–5.3)	0.249	
24-h urinary protein (mg/24 h)	2258.5 (380.0–5009.0)	641.5 (328.2–2429.2)	2685.0 (459.8–6280.0)	<0.001	***
Albumin subscore (0/2/4/6)	0.0 (0.0–2.0)	0.0 (0.0–0.0)	0.0 (0.0–2.0)	<0.001	***
Lymphocyte subscore (0/1/2/3)	1.0 (0.0–2.0)	0.0 (0.0–0.0)	1.0 (0.0–2.0)	<0.001	***
Cholesterol subscore (0/1/2/3)	1.0 (0.0–2.0)	0.0 (0.0–1.0)	2.0 (1.0–2.0)	<0.001	***
CONUT score (total)	3.0 (2.0–5.0)	1.0 (0.0–1.0)	4.0 (3.0–5.0)	<0.001	***
Urine protein (dipstick grade)	4: 131 (33.4%); 3: 98 (25.0%); 0: 86 (21.9%); 2: 43 (11.0%); 1: 33 (8.4%); 5: 1 (0.3%)	0: 26 (29.5%); 3: 21 (23.9%); 2: 15 (17.0%); 4: 15 (17.0%); 1: 11 (12.5%)	4: 116 (38.2%); 3: 77 (25.3%); 0: 60 (19.7%); 2: 28 (9.2%); 1: 22 (7.2%); 5: 1 (0.3%)	0.002	**
Urine occult blood (dipstick grade)	0: 239 (61.0%); 1: 49 (12.5%); 2: 47 (12.0%); 3: 41 (10.5%); 4: 16 (4.1%)	0: 57 (64.8%); 1: 13 (14.8%); 2: 12 (13.6%); 4: 4 (4.5%); 3: 2 (2.3%)	0: 182 (59.9%); 3: 39 (12.8%); 1: 36 (11.8%); 2: 35 (11.5%); 4: 12 (3.9%)	0.080	
Urine glucose (dipstick grade)	0: 108 (27.6%); 3: 86 (21.9%); 4: 84 (21.4%); 2: 56 (14.3%); 1: 42 (10.7%); 5: 16 (4.1%)	4: 25 (28.4%); 0: 19 (21.6%); 3: 15 (17.0%); 2: 14 (15.9%); 1: 9 (10.2%); 5: 6 (6.8%)	0: 89 (29.3%); 3: 71 (23.4%); 4: 59 (19.4%); 2: 42 (13.8%); 1: 33 (10.9%); 5: 10 (3.3%)	0.180	
Urine ketones (dipstick grade)	0: 366 (93.4%); 1: 15 (3.8%); 2: 10 (2.6%); 3: 1 (0.3%)	0: 77 (87.5%); 1: 7 (8.0%); 2: 3 (3.4%); 3: 1 (1.1%)	0: 289 (95.1%); 1: 8 (2.6%); 2: 7 (2.3%)	0.026	*

Data are presented as median (interquartile range) for continuous variables, and as frequency (percentage) for categorical variables. Nutritional status was defined by the CONUT score: normal, 0–1; malnutrition, ≥2. Values are derived from the first imputed dataset; missing laboratory values were handled by multiple imputation by chained equations (m = 20). Between-group comparisons used the Wilcoxon rank-sum test for continuous variables and the Pearson chi-square test for categorical variables. Urine dipstick grades are coded as: 0 = negative; 1 = trace (±); 2 = 1+; 3 = 2+; 4 = 3+; 5 = 4+. Sig column: *p < 0.05; **p < 0.01; ***p < 0.001. CO₂CP, carbon dioxide combining power; CONUT, Controlling Nutritional Status score; CRP, C-reactive protein; eGFR, estimated glomerular filtration rate (CKD-EPI equation); HbA1c, glycated haemoglobin; HDL-C, high-density lipoprotein cholesterol; IQR, interquartile range; LDL-C, low-density lipoprotein cholesterol.

Paradoxically, HbA1c was significantly lower in the malnutrition group than in the normal group (median 7.10% vs. 8.50%, p < 0.001), as was fasting glucose (*p* = 0.006). This inverse pattern is consistent with the phenomenon of renal resolution of hyperglycaemia in advanced DKD, whereby reduced renal insulin clearance, diminished appetite, and decreased carbohydrate intake collectively lower circulating glucose and glycated haemoglobin levels despite worsening overall metabolic status. Male sex showed a trend toward higher malnutrition prevalence (*p* = 0.054), while centre distribution did not differ significantly between groups (*p* = 0.15).

As expected from the construction of the CONUT score, the total score was strongly correlated with its three component variables in the first imputed dataset: albumin (Spearman *r* = −0.63), lymphocyte count (*r* = −0.60), and total cholesterol (*r* = −0.44). CONUT also showed a substantial inverse correlation with eGFR (r = −0.45), reflecting the well-established link between declining renal function and protein-energy wasting in DKD. The inverse correlation with HbA1c (*r* = −0.29) further corroborated the renal resolution of hyperglycaemia pattern observed in the bivariate comparisons. The association with eGFR indicates that, in DKD, CONUT may reflect renal disease severity and renal-related metabolic abnormalities in addition to nutritional risk.

Graded clinical deterioration across four CONUT categories was observed for most parameters (Supplementary Table S1). eGFR declined progressively from a median of 71 mL/min/1.73 m^2^ in the normal category to 47, 16, and 16 mL/min/1.73 m^2^ in the light, moderate, and severe categories, respectively. Albumin fell from 41.3 to 37.8, 31.5, and 25.6 g/L, and haemoglobin from 133 to 118, 104, and 94 g/L across the four categories. Centre 2 was disproportionately represented in the severe malnutrition category (36% vs. 9–20% in other categories, *p* = 0.020), underscoring the importance of adjusting for study centre in all regression analyses.

### 3.2 Tongue features across nutritional status groups

Across four CONUT categories, several tongue brightness and coating colour-moment features differed significantly (Supplementary Table S2). Edge tongue brightness (V) showed the strongest overall difference, with median values of 179, 183, 168, and 162 in the normal, light, moderate, and severe categories, respectively (*p* = 0.001), although the pattern was not strictly monotonic. Middle tongue brightness also differed across categories (medians: 196, 196, 193, and 188; *p* = 0.028), as did whole-tongue brightness (*p* = 0.020) and middle coating brightness (p = 0.028). Coating colour-moment features showed systematic shifts: first-order colour moments (mean intensity) for all three RGB channels decreased across categories (*p* = 0.003–0.005), while third-order colour moments (skewness) showed opposing directional trends. Edge tongue saturation (S) was highest in the severe malnutrition category (median 91 vs. 67 in the normal category, *p* = 0.038). By contrast, morphological features including tooth marks (*p* = 0.60), fissures (*p* = 0.70), coating thickness grade (*p* = 0.80), and coating ratio (*p* = 0.50) did not differ across nutritional categories, suggesting that CONUT-related tongue differences were primarily expressed in the colour and brightness dimensions rather than in macroscopic morphological features.

### 3.3 Confounder-adjusted partial correlations between tongue features and CONUT

**Figure 2:**
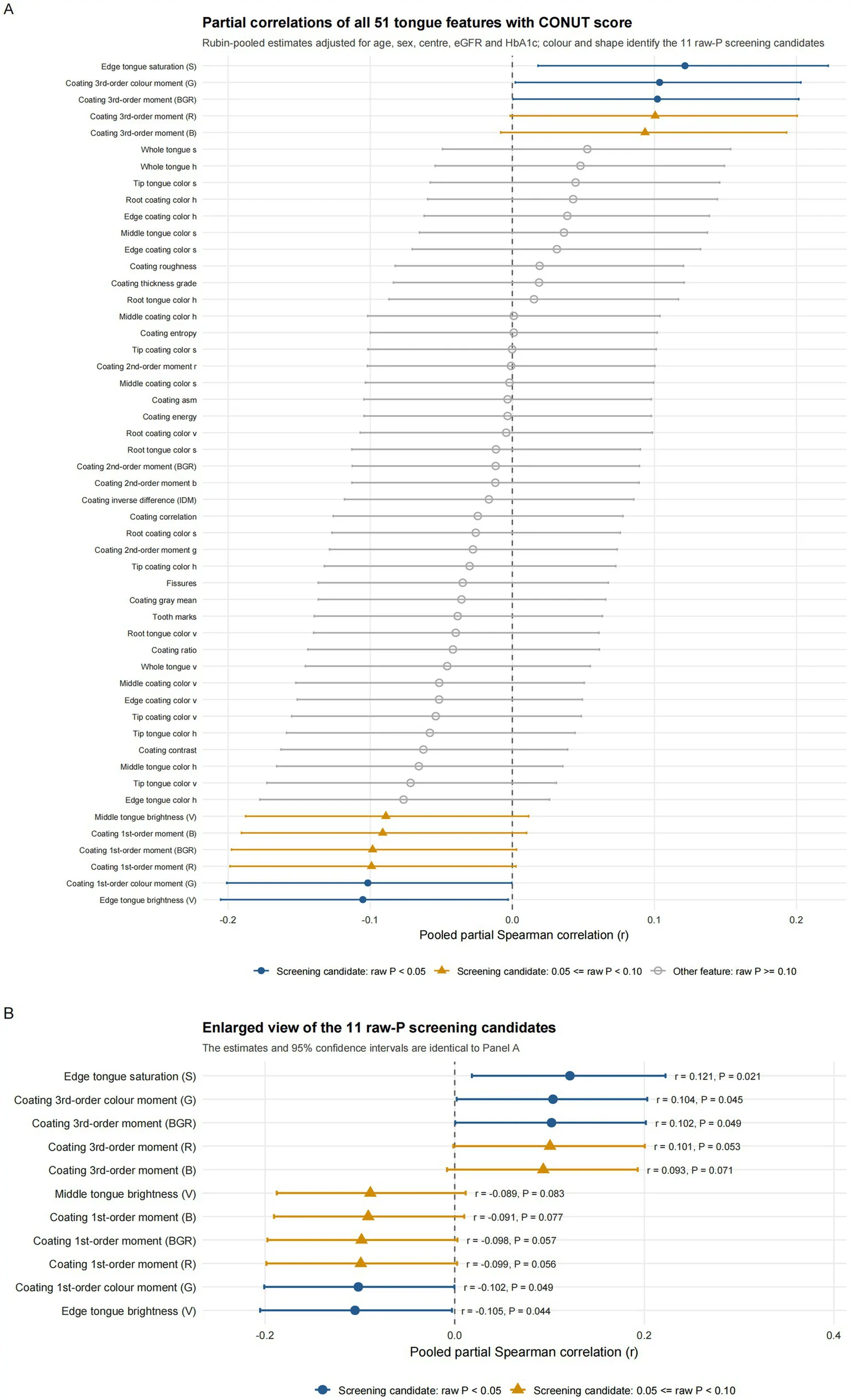
Rubin-pooled partial Spearman correlations between 51 tongue features and CONUT score. **(A)** Full view of all 51 tongue features. **(B)** Enlarged view of the 11 screening candidates with raw pooled *p* < 0.10, with individual *r* and *p* values annotated. Each point represents the Rubin-pooled partial Spearman correlation coefficient (*r*), with horizontal bars indicating 95% confidence intervals. Correlations were adjusted for age, sex, study centre, eGFR, and HbA1c across 20 imputed datasets, estimated via rank-based residualisation followed by Fisher z-transformation and Rubin pooling. Blue filled circles indicate raw *p* < 0.05, orange triangles indicate 0.05 ≤ raw *p* < 0.10, and grey open circles indicate raw *p* ≥ 0.10. Five features reached raw *p* < 0.05 and 11 reached raw *p* < 0.10; none survived Benjamini–Hochberg false discovery rate correction at *q* < 0.05 after testing all 51 tongue features. The vertical dashed line indicates zero. CONUT, Controlling Nutritional Status score; eGFR, estimated glomerular filtration rate; FDR, false discovery rate; HbA1c, glycated haemoglobin.

After adjusting for age, sex, study centre, eGFR, and HbA1c, Rubin-pooled partial Spearman correlations identified 11 tongue features with pooled *p* < 0.10, of which five reached nominal significance (*p* < 0.05; Figure 2; Supplementary Table S3). No feature survived BH FDR correction at the 0.05 threshold after adjustment for all 51 tongue variables simultaneously, consistent with the exploratory nature of this screening step.

The five nominally significant features comprised edge tongue saturation (*r* = +0.121, 95% CI 0.018 to 0.222, *p* = 0.021), edge tongue brightness (*r* = −0.105, 95% CI − 0.205 to −0.003, *p* = 0.044), coating third-order colour moment in the green channel (*r* = +0.104, *p* = 0.045), coating third-order BGR composite moment (*r* = +0.102, *p* = 0.049), and coating first-order colour moment in the green channel (*r* = −0.102, p = 0.049). Among the remaining six features with *p* < 0.10, middle tongue brightness showed an inverse association (*r* = −0.089, 95% CI − 0.188 to 0.012, *p* = 0.083), while the other candidates primarily comprised highly correlated first- and third-order coating colour moments.

The directional pattern of these associations was internally consistent: tongue features reflecting higher brightness or higher mean coating colour intensity (first-order moments) were inversely associated with CONUT, while features reflecting higher saturation or greater distributional skewness of coating colour (third-order moments) were positively associated. This convergent pattern—lower brightness, higher saturation, lower coating mean, higher coating skewness—suggests a coherent shift in tongue appearance accompanying increasing nutritional risk in DKD.

### 3.4 Adjusted single-feature regression analysis

Following collinearity pruning, the 11 candidate features were reduced to five cluster representatives. The pruning merged the four BGR-composite and single-channel coating colour-moment features into two clusters: coating first-order colour moment (green channel, representing the first-moment cluster) and coating third-order colour moment (green channel, representing the third-moment cluster). Edge tongue saturation, edge tongue brightness, and middle tongue brightness each formed independent single-feature clusters, yielding five representatives in total.

In Rubin-pooled univariate linear regression adjusted for age, sex, centre, eGFR, and HbA1c, all five cluster representatives reached BH FDR-adjusted *p* < 0.10 (Table 2). Middle tongue brightness showed the strongest association (*β* = −0.250 per SD, 95% CI − 0.461 to −0.038, *p* = 0.021), followed by coating first-order colour moment—green channel (*β* = −0.258, 95% CI − 0.480 to −0.036, *p* = 0.023), edge tongue brightness (*β* = −0.275, 95% CI − 0.517 to −0.033, *p* = 0.026), and edge tongue saturation (*β* = +0.241, 95% CI + 0.019 to +0.463, *p* = 0.033). Coating third-order colour moment—green channel showed a weaker but positive association (*β* = +0.192, 95% CI − 0.017 to +0.400, *p* = 0.071). The direction of all associations was consistent with the partial correlation findings: higher brightness and higher mean coating colour intensity were associated with lower CONUT scores, whereas higher edge saturation and greater coating colour skewness were associated with higher CONUT scores.

**Table 2:** Rubin-pooled adjusted single-feature linear regression of cluster-representative tongue features against CONUT score.

Tongue feature	β (per SD)	SE	95% CI	*p* value	P (FDR-adj)
Middle tongue brightness (V)	−0.250	0.108	−0.461 to −0.038	0.021	0.042
Coating 1st-order colour moment, G channel	−0.258	0.113	−0.480 to −0.036	0.023	0.042
Edge tongue brightness (V)	−0.275	0.124	−0.517 to −0.033	0.026	0.042
Edge tongue saturation (S)	0.241	0.113	0.019 to 0.463	0.033	0.042
Coating 3rd-order colour moment, G channel	0.192	0.106	−0.017 to 0.400	0.071	0.071

β coefficients represent the change in CONUT score per one standard deviation increase in the tongue feature, estimated from Rubin-pooled adjusted single-feature linear regression across 20 imputed datasets. Each feature is the cluster representative selected after hierarchical collinearity pruning (|r| ≥ 0.80). All models were adjusted for age, sex, study centre, eGFR, and HbA1c. Tongue features were z-score standardised using the mean and standard deviation of the first imputed dataset. All five features reached Benjamini–Hochberg FDR-adjusted *p* < 0.10. CI, confidence interval; CONUT, Controlling Nutritional Status score; eGFR, estimated glomerular filtration rate; FDR, false discovery rate; HbA1c, glycated haemoglobin; HSV, hue-saturation-value; P (FDR-adj), Benjamini–Hochberg FDR-adjusted p-value; SD, standard deviation; SE, standard error.

### 3.5 Multivariable regression analysis

**Figure 3:**
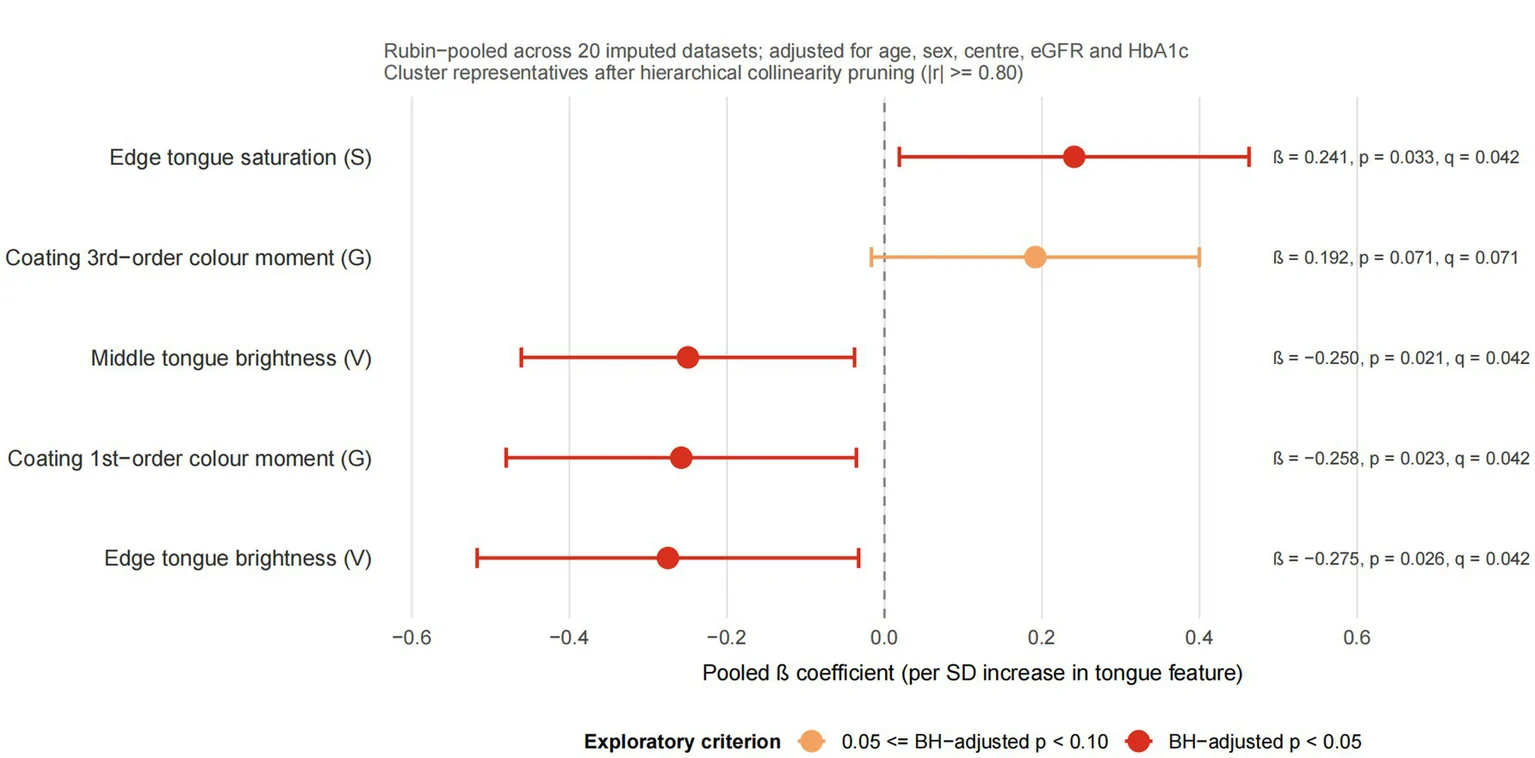
Rubin-pooled single-feature linear regression of five cluster-representative tongue features against CONUT score. Each point represents the Rubin-pooled regression coefficient (*β*) for one z-score-standardised tongue feature, with horizontal bars indicating 95% confidence intervals. Models were adjusted for age, sex, study centre, eGFR, and HbA1c across 20 imputed datasets. All five features were selected after hierarchical collinearity pruning at |*r*| ≥ 0.80 and met the exploratory criterion of Benjamini–Hochberg-adjusted *p* < 0.10. Red points indicate BH-adjusted *p* < 0.05, and orange points indicate 0.05 ≤ BH-adjusted *p* < 0.10. The vertical dashed line indicates zero. *β* coefficients represent the change in CONUT score per one standard deviation increase in the tongue feature. CONUT, Controlling Nutritional Status score; eGFR, estimated glomerular filtration rate; HbA1c, glycated haemoglobin; SD, standard deviation.

All five cluster representatives were entered into the pooled multivariable linear regression model. Compared with the single-feature estimates, the associations were substantially attenuated for edge tongue brightness (*β* = +0.067, *p* = 0.724), coating first-order colour moment in the green channel (*β* = −0.087, *p* = 0.677), and coating third-order colour moment in the green channel (*β* = +0.069, *p* = 0.689). By contrast, edge tongue saturation (*β* = +0.269, 95% CI − 0.023 to +0.560, *p* = 0.071) and middle tongue brightness (*β* = −0.234, 95% CI − 0.495 to +0.026, *p* = 0.078) retained directionally consistent, borderline associations in the joint model (Table 3). Neither feature reached the conventional *p* < 0.05 threshold, and these findings were therefore considered exploratory. The direction and magnitude of all five associations are summarised in Figure 3.

**Table 3:** Rubin-pooled multivariable linear regression of tongue features against CONUT score.

Section	Term	Adjusted β	SE	95% CI	*p* value
Tongue features	Middle tongue brightness (V)	−0.234	0.133	−0.495 to 0.026	0.078
Tongue features	Coating 1st-order colour moment (G)	−0.087	0.208	−0.494 to 0.321	0.677
Tongue features	Edge tongue brightness (V)	0.067	0.190	−0.305 to 0.439	0.724
Tongue features	Edge tongue saturation (S)	0.269	0.149	−0.023 to 0.560	0.071
Tongue features	Coating 3rd-order colour moment (G)	0.069	0.172	−0.269 to 0.406	0.689
Covariates	Age (per year)	0.021	0.009	0.004 to 0.039	0.015
Covariates	Sex (male vs. female)	0.692	0.234	0.232 to 1.151	0.003
Covariates	Centre 2 vs. centre 1	0.194	0.367	−0.527 to 0.914	0.598
Covariates	eGFR (per mL/min/1.73 m^2^)	−0.029	0.003	−0.035 to −0.022	<0.001
Covariates	HbA1c (per 1%)	−0.104	0.057	−0.216 to 0.008	0.067

Estimates were obtained from a single Rubin-pooled multivariable model simultaneously including all five cluster-representative tongue features and covariates (age, sex, study centre, eGFR, and HbA1c) across 20 imputed datasets. For tongue features, β coefficients represent the change in CONUT score per one standard deviation increase; coefficients for continuous covariates represent the change per unit increase, and categorical coefficients represent the stated contrast. Tongue features were z-score standardised. Variance inflation factors for all model terms ranged from 1.09 to 4.01 (Supplementary Table S4), indicating no severe multicollinearity. CI, confidence interval; CONUT, Controlling Nutritional Status score; eGFR, estimated glomerular filtration rate; HbA1c, glycated haemoglobin; HSV, hue-saturation-value; SD, standard deviation; SE, standard error; VIF, variance inflation factor.

Among covariates, eGFR showed the strongest association with CONUT in the full model (*β* = −0.0286 per mL/min/1.73 m^2^, 95% CI − 0.035 to −0.022, *p* < 0.001). Male sex (*β* = +0.692, *p* = 0.003) and older age (*β* = +0.021 per year, *p* = 0.015) were also associated with higher CONUT scores, while HbA1c showed a borderline inverse association (*β* = −0.104, *p* = 0.067). Study centre was not independently associated with CONUT (*β* = +0.194, *p* = 0.598). Variance inflation factors ranged from 1.09 to 4.01, indicating no severe multicollinearity.

### 3.6 Sensitivity analyses

**Figure 4:**
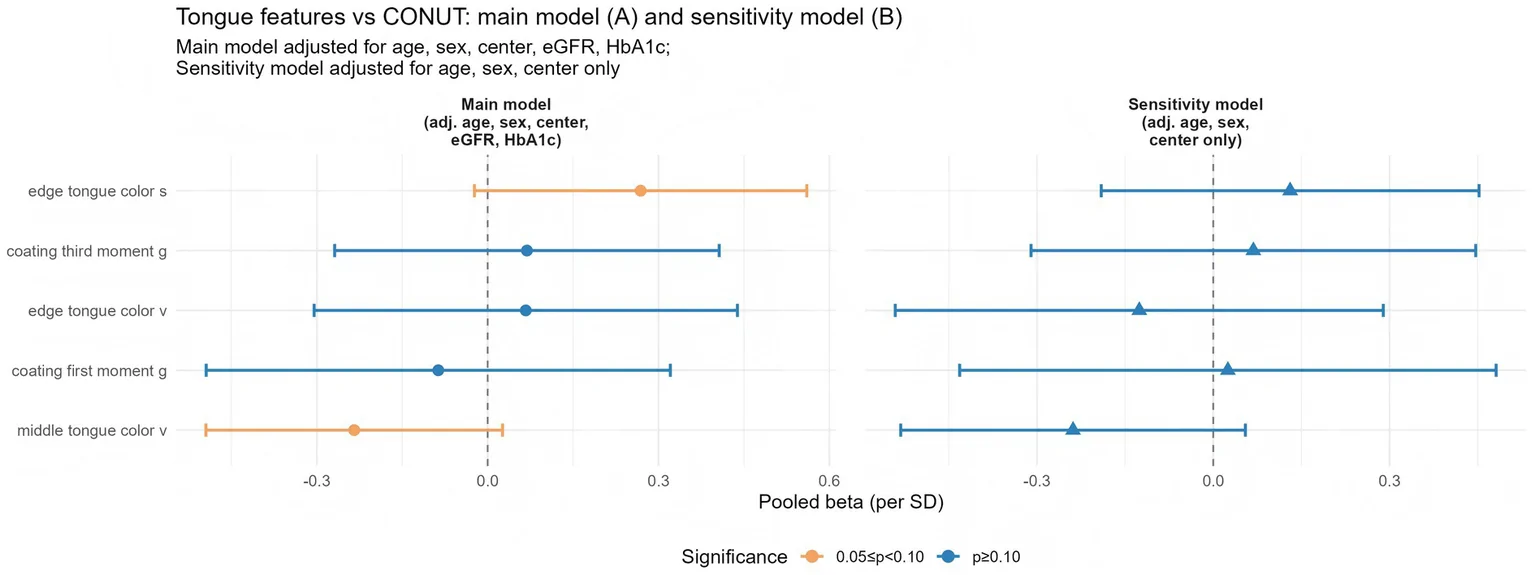
Multivariable regression of tongue features against CONUT score: Primary model **(A)** and sensitivity model **(B)**. (A) Primary multivariable model adjusted for age, sex, study centre, eGFR, and HbA1c. **(B)** Sensitivity model adjusted for age, sex, and study centre only, with eGFR and HbA1c excluded. Each point represents the Rubin-pooled regression coefficient (*β*) for one z-score-standardised tongue feature, with horizontal bars indicating 95% confidence intervals. All models were pooled across 20 imputed datasets. Point colour indicates the level of raw statistical evidence: Orange, 0.05 ≤ *p* < 0.10; blue, *p* ≥ 0.10. In the primary model, edge tongue saturation and middle tongue brightness retained borderline associations with CONUT (*p* = 0.071 and *p* = 0.078, respectively). Excluding eGFR and HbA1c attenuated the edge tongue saturation coefficient from +0.269 to +0.131, consistent with the suppressor pattern involving eGFR, whereas the middle tongue brightness coefficient remained similar in magnitude (−0.234 to −0.239) but imprecise. The vertical dashed line indicates zero. CONUT, Controlling Nutritional Status score; eGFR, estimated glomerular filtration rate; HbA1c, glycated haemoglobin; SD, standard deviation.

When eGFR and HbA1c were removed from the covariate set, the associations of edge tongue saturation and middle tongue brightness with CONUT changed in contrasting ways (Table 4; Figure 4). Middle tongue brightness remained stable across adjustment models (*β* = −0.239 in the demographic-only model vs. − 0.234 in the primary model, a 1.8% change), suggesting that the estimated association was largely insensitive to adjustment for renal function and glycaemic control. Edge tongue saturation, by contrast, attenuated substantially when eGFR and HbA1c were removed (*β* = +0.131, *p* = 0.425), approximately halving the effect size observed in the primary model (*β* = +0.269). Middle tongue brightness approached the exploratory threshold in both models (primary model *p* = 0.078; demographic-only model *p* = 0.111), whereas edge tongue saturation was not significant in the demographic-only model, highlighting the sensitivity of its estimated association to renal adjustment.

**Table 4:** Sensitivity analyses: tongue feature coefficients under alternative covariate specifications.

Tongue feature	Model A: β (95% CI)	Model A: P	Model B: β (95% CI)	Model B: P	Model C: β (95% CI)	Model C: P	Change A → C (%)	Stability
Middle tongue brightness (V)	−0.234 (−0.495 to 0.026)	0.078	−0.239 (−0.532 to 0.055)	0.111	−0.204 (−0.463 to 0.055)	0.122	12.9%	Modest attenuation (10–25%)
Edge tongue saturation (S)	0.269 (−0.023 to 0.560)	0.071	0.131 (−0.191 to 0.452)	0.425	0.242 (−0.047 to 0.531)	0.101	−9.9%	Stable (<10% change)
Coating 1st-order colour moment, G channel	−0.087 (−0.494 to 0.321)	0.677	0.025 (−0.432 to 0.481)	0.915	−0.097 (−0.501 to 0.306)	0.636	−12.3%	Modest change (10–25%)
Coating 3rd-order colour moment, G channel	0.069 (−0.269 to 0.406)	0.689	0.068 (−0.310 to 0.447)	0.724	0.082 (−0.253 to 0.417)	0.629	19.7%	Modest change (10–25%)
Edge tongue brightness (V)	0.067 (−0.305 to 0.439)	0.724	−0.126 (−0.541 to 0.289)	0.552	0.056 (−0.311 to 0.424)	0.764	−15.7%	Modest attenuation (10–25%)

Model A: primary multivariable model adjusted for age, sex, study centre, eGFR, and HbA1c. Model B: sensitivity model adjusted for age, sex, and study centre only (eGFR and HbA1c excluded). Model C: Model A covariate set with additional adjustment for log-transformed 24-h urinary protein [log(1 + U24h_protein)]. β coefficients are Rubin-pooled across 20 imputed datasets and represent the change in CONUT score per one standard deviation increase in the tongue feature. Percentage change is calculated as (β_C − β_A) / |β_A| × 100%. CI, confidence interval; CONUT, Controlling Nutritional Status score; eGFR, estimated glomerular filtration rate; HbA1c, glycated haemoglobin; HSV, hue-saturation-value; SD, standard deviation; U24h_protein, 24-h urinary protein.

**Figure 5:**
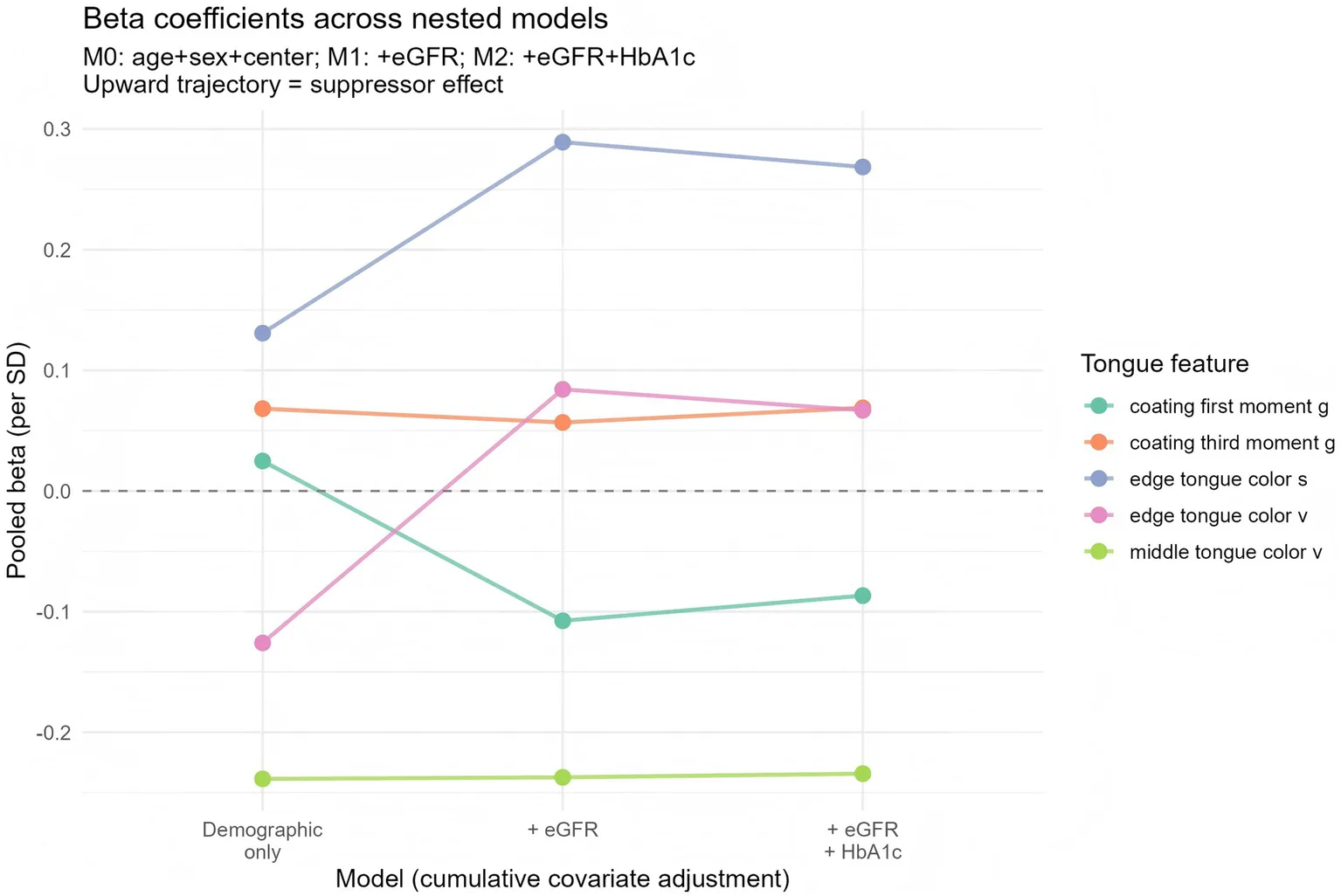
Nested model analysis: Trajectories of tongue feature coefficients across progressive covariate adjustment. Rubin-pooled regression coefficients (*β*, per *SD*) for each of the five tongue features are plotted across three progressively adjusted models: Model 1 (age, sex, and study centre only), Model 2 (Model 1 plus *eGFR*), and Model 3 (Model 2 plus *HbA1c*; the primary model). Lines connect estimates for the same feature across models. The edge tongue saturation coefficient increased from +0.131 in Model 1 to +0.289 after adjustment for *eGFR* in Model 2 and remained +0.269 in Model 3. This trajectory is consistent with a suppressor pattern in which adjustment for *eGFR* reveals a stronger positive tongue–*CONUT* association estimate. The near-horizontal trajectory of middle tongue brightness (*β* = −0.239, −0.237, and −0.234 in Models 1, 2, and 3, respectively) indicates comparative stability of the coefficient magnitude across covariate sets, although its confidence intervals included zero. The horizontal dashed line indicates zero. All estimates were pooled across 20 imputed datasets. *CONUT*, Controlling Nutritional Status score; *eGFR*, estimated glomerular filtration rate; *HbA1c*, glycated haemoglobin; *SD*, standard deviation.

To characterise the role of eGFR and HbA1c more precisely, we fitted three progressively adjusted models for each tongue feature: Model 1 (age, sex, and centre only), Model 2 (adding eGFR), and Model 3 (adding HbA1c; the primary model). For edge tongue saturation, the coefficient increased markedly from *β* = +0.131 in Model 1 to *β* = +0.289 in Model 2, representing a 121% increase in magnitude, and then decreased modestly to *β* = +0.269 in Model 3 (Figure 5). This trajectory is consistent with the statistical pattern of suppression: adjustment for eGFR removed shared variation and revealed a stronger positive estimate for the association between edge tongue saturation and CONUT. For middle tongue brightness, the coefficient remained stable across all three models (*β* = −0.239, −0.237, and −0.234, respectively), supporting the interpretation that its estimated association was not materially driven by renal function or glycaemic adjustment. Among the remaining three features, coating first-order colour moment and edge tongue brightness showed sign reversals across models, whereas coating third-order colour moment remained close to zero. These patterns suggest that their single-feature associations did not represent stable independent contributions after mutual adjustment for the other tongue features and clinical covariates.

Adding log-transformed 24-h urinary protein to the full covariate set produced coefficient changes below 20% for all five tongue features (Table 4). Edge tongue saturation decreased by 9.9% to *β* = +0.242 (95% CI − 0.047 to +0.531, *p* = 0.101), whereas middle tongue brightness decreased in magnitude by 12.9% to *β* = −0.204 (95% CI − 0.463 to +0.055, *p* = 0.122). Proteinuria therefore did not materially change the estimated directions or magnitudes, although neither association reached statistical significance after additional adjustment.

### 3.7 Ordinal logistic regression

In Rubin-pooled ordinal logistic regression with CONUT nutritional category as the ordered outcome, edge tongue saturation was the only tongue feature with a borderline association (OR = 1.29 per SD increase, 95% CI 0.96 to 1.71, pooled *p* = 0.086; Supplementary Table S5). The direction and magnitude of this estimate were consistent with the primary linear model (β = +0.269), and the widening of the confidence interval relative to the first-imputation estimate (OR = 1.35, 95% CI 1.02 to 1.78, *p* = 0.033) reflects the propagation of between-imputation uncertainty through Rubin pooling. Middle tongue brightness showed a directionally consistent but non-significant inverse association (OR = 0.86, 95% CI 0.67 to 1.10, *p* = 0.216). The remaining three features showed negligible associations in the ordinal model (all *p* > 0.66), consistent with their attenuation in the primary joint linear regression. No tongue feature survived BH FDR correction in the ordinal model. Taken together, the ordinal logistic results support edge tongue saturation as the most consistent signal across the primary linear and ordinal modelling frameworks, although the pooled association remained below the conventional significance threshold.

### 3.8 Subgroup and interaction analyses

In eGFR-stratified models, middle tongue brightness (*β* = −0.345, 95% CI − 0.657 to −0.034, *p* = 0.030) and edge tongue saturation (*β* = +0.395, 95% CI + 0.002 to +0.787, *p* = 0.049) were associated with CONUT among patients with eGFR ≥45 mL/min/1.73 m^2^, whereas neither feature was significant among those with eGFR <45 mL/min/1.73 m^2^. However, formal continuous eGFR interaction tests were non-significant for middle tongue brightness (p for interaction = 0.611) and edge tongue saturation (*p* for interaction = 0.567), and no eGFR interaction survived BH correction. Thus, differences between the eGFR strata did not establish effect modification.

In sex-stratified models, middle tongue brightness was inversely associated with CONUT among men (*β* = −0.312, 95% CI − 0.594 to −0.030, p = 0.030) but not among women (*β* = +0.193, *p* = 0.565). The raw interaction *p* value was 0.045 for middle tongue brightness, while edge tongue brightness had a raw interaction p value of 0.034. However, both had BH-adjusted *p* = 0.113, and no tongue feature-by-sex interaction remained significant after correction. These subgroup findings were therefore considered exploratory.

## 4 Discussion

This dual-centre cross-sectional study systematically examined the associations between objectively quantified tongue features and the CONUT nutritional risk score in 392 patients with DKD. Three principal findings emerge. First, the prevalence of CONUT-defined malnutrition was high, affecting 77.6% of patients, with moderate-to-severe malnutrition present in more than one quarter. Second, after adjustment for age, sex, study centre, eGFR, and HbA1c, edge tongue saturation and middle tongue brightness retained modest, borderline associations with CONUT in the primary joint model (*β* = +0.269 and −0.234, respectively), although neither reached the conventional *p* < 0.05 threshold. The directions of these estimates were generally maintained in the linear sensitivity analyses, but their confidence intervals included zero. Third, nested model analysis showed a statistical suppression pattern for eGFR in the edge tongue saturation–CONUT association, whereas the coefficient for middle tongue brightness remained comparatively stable across adjustment models. Exploratory subgroup analyses did not provide definitive evidence of effect modification after multiple-comparison correction. Taken together, these findings suggest a modest and hypothesis-generating correspondence between tongue phenotype and CONUT-defined nutritional risk in DKD.

### 4.1 Nutritional burden in DKD and the clinical context of CONUT

The observation that 77.6% of patients in this cohort had a CONUT score of ≥2 is consistent with a growing body of evidence documenting the high prevalence of PEW and malnutrition in CKD and DKD populations. In a meta-analysis of contemporary observational studies coordinated by the International Society of Renal Nutrition and Metabolism, Carrero et al. reported that PEW prevalence in non-dialysis CKD stages 3–5 ranged from 11 to 54% across studies, rising to a 25th–75th percentile range of 28–54% among the 16,434 maintenance dialysis patients included, underscoring that PEW is a common phenomenon across the full spectrum of kidney disease (28). Although the full diagnostic criteria for PEW extend beyond biochemical parameters, the high prevalence of protein and lipid metabolic derangements in DKD creates a population in which biochemical nutritional screening tools such as the CONUT score are clinically meaningful and operationally practical. The CONUT score, originally validated by de Ulíbarri et al. in 2005 as a bedside nutritional screening tool requiring only three routine laboratory parameters, has subsequently been applied to diverse clinical populations and shown prognostic utility in patients with CKD and cardiovascular comorbidities (7). In DKD specifically, higher CONUT scores have been independently associated with increased all-cause mortality and adverse renal outcomes in cohort studies, supporting its prognostic utility in this population (6).

The paradoxical inverse relationship between HbA1c and CONUT score observed in this cohort—where more malnourished patients had lower HbA1c—is not unique to our study and has been previously described in advanced DKD. The mechanisms underlying this phenomenon are multifactorial: reduced erythrocyte lifespan in anaemia shortens the time window for haemoglobin glycation; decreased renal insulin clearance augments insulin availability and lowers glucose; and reduced dietary carbohydrate intake secondary to uraemic anorexia further suppresses glycaemia (29, 30). Clinically, this pattern underscores the risk of using HbA1c as the sole indicator of glycaemic management in patients with advanced DKD, and the importance of nutritional assessment as a complementary dimension of disease monitoring that HbA1c cannot capture.

The finding that eGFR showed the strongest covariate association with CONUT in the full multivariable model (*β* = −0.0286 per mL/min/1.73 m^2^, *p* < 0.001) aligns with established pathophysiology: progressive glomerulosclerosis and tubular dysfunction in DKD contribute to uraemic inflammation, anorexia, urinary protein loss, and impaired protein and lipid metabolism, all of which may influence serum albumin, lymphocyte count, and total cholesterol (3). Consequently, CONUT should not be interpreted as a measure of nutritional status alone in DKD; it may also capture aspects of renal disease severity and the associated metabolic disturbance, even after statistical adjustment for eGFR. Male sex was independently associated with higher CONUT scores in our cohort. While this association has not been specifically reported in prior DKD nutritional screening studies, it is biologically plausible: male patients may be predisposed to greater nutritional depletion through higher baseline muscle protein turnover, differential susceptibility to uraemic anorexia, and a higher prevalence of cardiovascular comorbidities that increase metabolic demand. Whether this sex difference in CONUT scores reflects a genuine difference in nutritional vulnerability or is partly attributable to differences in body composition and reference ranges for the CONUT component variables warrants examination in larger, sex-stratified cohorts.

### 4.2 Tongue phenotype as a window into nutritional status: integrating TCM theory and biomedical mechanisms

The two tongue features that most consistently reflected CONUT-defined nutritional risk in this study—edge tongue saturation and middle tongue brightness—map onto distinct dimensions of tongue appearance that carry interpretable meaning in both TCM diagnostic theory and contemporary biomedical understanding of DKD pathophysiology.

Middle tongue brightness (V in the HSV colour model) showed a comparatively stable coefficient across the nested linear models, changing only from *β* = −0.239 to −0.234 after adding eGFR and HbA1c, although additional proteinuria adjustment attenuated the coefficient to −0.204. In the HSV model, the V channel quantifies luminous reflectance of the tongue surface, with lower values corresponding to a darker, less luminous appearance. Reduced tongue brightness is consistent with the TCM concept of a pale or dusky tongue body, which is classically associated with deficiency of qi and blood and insufficient nourishment of the tongue tissue. From a biomedical perspective, the tongue mucosa is a highly vascularised tissue whose colour and luminosity are directly influenced by haemoglobin concentration and microvascular perfusion. In DKD, the combination of normocytic or microcytic anaemia—driven by reduced erythropoietin synthesis, iron deficiency, and chronic inflammation—and hypoalbuminaemia-related tissue oedema reduces the haemoglobin-dependent red reflectance and overall luminance of the tongue surface (31, 32). The relative stability of the middle tongue brightness estimate after eGFR adjustment is noteworthy and suggests that this feature may capture downstream manifestations of nutritional depletion, such as anaemia and hypoalbuminaemia, that are not fully represented by glomerular filtration rate alone.

Edge tongue saturation (S in the HSV colour model) exhibited a different and methodologically more complex pattern. The S channel quantifies the purity or intensity of the chromatic signal, with higher values corresponding to a more vivid, deeply coloured tongue edge. In TCM, the tongue edges are attributed to the liver and gallbladder meridians, and a reddened or hypersaturated tongue edge is associated with either liver qi stagnation transforming into fire or yin deficiency with endogenous heat, both of which may manifest as increased colour saturation at the tongue margins—patterns that in the context of advanced DKD may reflect chronic systemic inflammation, oxidative stress, and microcirculatory dysfunction (33). Biomedically, increased edge saturation in malnourished patients may correspond to compensatory vasodilation or localised inflammatory hyperaemia at the tongue margins, where microvascular responses to nutritional stress and uraemic toxin accumulation are more readily observable than at the thicker, more keratinised central tongue surface (34).

Importantly, the four-category comparison showed that edge tongue saturation was highest in the severe malnutrition category (median S = 91 versus 67 in the normal category), while middle tongue brightness was lower in the severe category than in the normal category, although the across-category brightness pattern was not strictly monotonic. This combination of lower central brightness and higher edge saturation in the severe category is consistent with a composite tongue pattern in which central pallor (qi-blood deficiency) coexists with peripheral hypersaturation (liver-blood stasis and endogenous heat), a pattern that experienced TCM clinicians associate with chronic organ depletion superimposed on metabolic obstruction. To our knowledge, this is the first study to quantify this composite tongue phenotype in a DKD population using objective colorimetric methods, and to link it systematically to a validated nutritional screening tool.

### 4.3 The suppressor effect of eGFR: methodological implications and pathophysiological interpretation

The nested model analysis revealed a clear statistical suppression pattern for eGFR in the edge tongue saturation–CONUT association: the coefficient for edge saturation increased by 121% (from *β* = +0.131 to *β* = +0.289) after adjustment for eGFR. A suppressor variable is defined as a covariate that, when added to a regression model, increases the coefficient of another predictor by removing outcome-irrelevant variance shared between the predictor and the suppressor (27). In the present analysis, adding eGFR revealed a stronger positive estimate for the association between edge tongue saturation and CONUT, which was subsequently only modestly attenuated after adding HbA1c (β = +0.269). This coefficient trajectory is consistent with statistical suppression, although the cross-sectional data cannot establish the biological pathway responsible for this pattern.

This finding has important implications for the design of future studies investigating tongue–biomarker associations in DKD. Bivariate analyses or models that omit renal function adjustment may underestimate the strength of certain tongue–nutritional associations, particularly those involving tongue edge colour, which appears to be especially sensitive to the competing influences of uraemic microvascular injury and liver-blood depletion. Our results suggest that renal adjustment is not merely a statistical formality but an important analytical consideration for isolating the nutritional signal embedded in tongue edge appearance.

The contrast between the suppressor behaviour of eGFR for edge saturation and the relative stability of middle tongue brightness across renal-adjustment models further illuminates the anatomical and physiological specificity of tongue regions in reflecting different pathophysiological processes The tongue centre, in TCM theory attributed to the spleen and stomach, is the primary locus of digestive and absorptive function; its brightness decline with worsening nutritional status may reflect a more direct consequence of gastrointestinal dysfunction, reduced nutrient absorption, and systemic protein-energy depletion that is relatively upstream of renal filtration decline. The tongue edges, attributed to the liver, are more sensitive to haemodynamic and inflammatory signals that are intertwined with—but not reducible to—glomerular filtration. This anatomical specificity of tongue regions for distinct pathophysiological axes in DKD represents a potentially valuable framework for precision syndrome differentiation in clinical practice.

### 4.4 Coating colour moments: a biologically plausible but statistically attenuated signal

The coating first-order and third-order colour moments in the green channel met the prespecified exploratory BH-adjusted threshold of q < 0.10 in single-feature regression but lost their associations almost entirely in the joint multivariable model. This attenuation reflects primarily the statistical competition among correlated features rather than the absence of a biological signal. First-order colour moments represent mean coating colour intensity, and their decrease across CONUT categories suggests a systematic shift of the tongue coating toward a lower-intensity, paler, or more dilute appearance with worsening nutritional status—a finding consistent with the TCM concept of a thin, pale coating associated with spleen-stomach qi deficiency and insufficient transformation and transportation of food essence. Third-order moments (colour skewness) reflect the distributional asymmetry of coating pixel intensities; their increase may indicate the emergence of local coating inhomogeneity, patchy denudation, or uneven colour distribution—features that in TCM parlance correspond to a peeled or partially peeled coating, which is classically associated with yin deficiency and gastric injury in chronic disease (35, 36).

From a microbiological standpoint, the tongue coating is predominantly composed of desquamated epithelial cells, saliva components, food debris, and oral microbiota. Malnutrition-related changes in salivary flow rate, oral immune defence, and epithelial cell turnover alter coating texture and colour distribution (37, 38). In the context of DKD, uraemic compounds including urea, creatinine, and indoxyl sulphate are secreted into the oral cavity and may interact with the coating microbiome, generating ammonia and other volatile compounds that alter coating colour moments in ways that are not simply reducible to renal function (39). However, because coating colour moments were moderately correlated with edge saturation and middle brightness in the multivariable model—reflecting a shared biological substrate of systemic nutritional depletion that manifests simultaneously as changes in tongue body colour, tongue edge chromatic intensity, and coating colour distribution—their independent statistical contribution to CONUT prediction was largely captured by the two tongue body colour features, leading to attenuation of their multivariable coefficients. Future studies that specifically target coating texture and colour heterogeneity as primary exposures, with adequate sample sizes to support stable multivariable estimation, are warranted to clarify the independent nutritional information carried by coating colour moments.

### 4.5 Comparison with existing literature on objective tongue assessment in DKD

The present study builds upon a small but emerging body of literature that has applied objective, AI-assisted tongue imaging to characterise tongue phenotypes in DKD populations. Two previously published studies from our group are directly relevant to contextualising the current findings.

In our earlier investigation of the relationship between the atherogenic index of plasma (AIP) and objective tongue features in DKD patients, we identified that higher AIP—reflecting dyslipidaemia and increased cardiovascular risk—was associated with changes in tongue tip and tongue centre colour saturation and brightness, as well as coating colour hue and saturation (20). The directional consistency between those findings and the present results is notable: in both studies, tongue colour saturation and brightness in the central and tip regions showed associations with metabolic adversity, suggesting that the tongue centre and tip may serve as a broad barometer of metabolic dysregulation in DKD rather than being specific to any single pathological pathway. However, the AIP study focused on a lipid-related cardiovascular risk index and did not examine nutritional status per se, leaving open the question of whether tongue changes associated with dyslipidaemia and those associated with protein-energy wasting represent overlapping or distinct tongue phenotypes. The current study begins to address this question by showing that features from both the middle and edge regions retained the clearest exploratory signals after joint adjustment, suggesting potential regional specificity in tongue-to-biomarker mapping.

Our study of tongue features across DKD stages stratified by damp-heat syndrome further informs the interpretation of the current results (21). In that study, conducted in 134 DKD patients with damp-heat syndrome across CKD G3–G5 stages, we found that tongue tip and edge pallor decreased progressively from G3 to G5, while tongue centre colour hue and coating brightness increased in the G4 group relative to G5. Those findings pointed to a progressive shift in tongue colour phenotype with advancing CKD stage, with the most severe stage (G5) showing features consistent with qi-blood deficiency and yin depletion. In the present study, the association between lower middle tongue brightness and higher CONUT scores after adjustment for eGFR extends these observations by suggesting that the tongue centre brightness signal may reflect a nutritional dimension beyond its relationship with renal function alone. The convergence of evidence from syndrome-stratified studies and the present biomarker-anchored analysis supports the biological plausibility of middle tongue brightness as a candidate nutritional indicator for further investigation in DKD.

Studies applying objective tongue imaging to chronic kidney disease have predominantly focused on eGFR-based staging and renal function characterisation. Chung et al. developed a tongue diagnosis index for CKD and demonstrated that quantitative tongue colour and morphological parameters correlated with CKD stage, supporting the feasibility of tongue phenotyping as a non-invasive tool for renal function assessment (14). More recently, a recent perspective proposed “tongue age” as a multidimensional indicator integrating tongue-coating microbiome profiles with digital imaging phenotypes to characterise biological ageing and systemic disease risk (40). This framework broadens objective tongue assessment beyond individual colour or texture parameters and supports its potential use in multidimensional health-risk characterisation, while also emphasising the need for standardisation and external validation. However, whether tongue features carry information about nutritional status beyond their association with eGFR has not been examined. The present study addresses this gap directly: by adjusting for eGFR as a covariate rather than treating it as the outcome, our findings suggest that residual tongue variation after renal adjustment may carry information related to nutritional risk—a dimension of DKD pathophysiology that prior tongue imaging studies have not explored.

### 4.6 Clinical implications and translational value

The findings of this study carry several practical implications for the clinical management of DKD, particularly in the context of integrative medicine settings where TCM and Western medicine are practised in conjunction.

A broader mechanism-oriented framework is also emerging across diabetes-related end-organ complications. For example, a recent perspective reviewed the potential effects of TCM interventions in diabetic retinopathy through pathways including PI3K–Akt and AGE–RAGE signalling (41). Given the shared involvement of chronic inflammation, oxidative stress, and microvascular injury in diabetic retinopathy and DKD, this work provides a parallel perspective on the integration of TCM concepts with modern mechanistic research, although it does not directly validate tongue phenotyping as a clinical screening tool.

First, the high prevalence of CONUT-defined malnutrition (77.6%) in this cohort underscores the need for routine nutritional screening in DKD clinical practice. Current guidelines from the KDIGO and the Global Leadership Initiative on Malnutrition (GLIM) recommend regular nutritional assessment in CKD patients, yet implementation remains inconsistent in real-world practice, partly because nutritional screening tools require blood tests that may not be ordered at every clinic visit (42, 43). The CONUT score, requiring only albumin, lymphocyte count, and total cholesterol—all of which are included in the routine biochemical panels of most DKD outpatient visits—is particularly well suited for widespread implementation in this population. If validated in prospective studies, tongue appearance might provide a rapid, non-invasive prompt for clinicians to consider formal nutritional assessment; however, the present findings do not support the use of tongue features as a stand-alone screening test.

Second, the suppression analysis has implications for the analytical interpretation of tongue-based nutritional signals. Our results indicate that the estimated association between edge tongue saturation and CONUT was substantially weaker when renal function was omitted from the model. This finding does not support individual-level risk reclassification based on tongue appearance, but suggests that eGFR should be considered in future studies developing or validating tongue-based nutritional assessment models in DKD.

Third, the regional specificity observed in this study—tongue centre brightness reflecting nutritional depletion relatively independently of renal function, and tongue edge saturation requiring renal adjustment to reveal its nutritional signal—provides a practical framework for tongue-based triage. In resource-limited settings where laboratory testing is infrequent, middle tongue brightness and edge tongue saturation may warrant further evaluation as components of a preliminary nutritional-risk assessment approach, provided that their incremental value beyond renal function and routine clinical information is prospectively validated. AI-assisted tongue imaging systems such as the YZAI-02 platform used in this study can generate these quantitative parameters in real time at the point of care, making this a technically feasible approach for integration into routine DKD follow-up.

### 4.7 Limitations

Several limitations warrant consideration. First, the cross-sectional design precludes causal inference; the observed associations may reflect shared pathophysiological determinants, and longitudinal studies are needed to establish the temporal relationship between tongue changes and nutritional status. Second, CONUT is not a pure measure of nutritional depletion in DKD. Albumin, lymphocyte count, and total cholesterol may all be directly affected by renal dysfunction, inflammation, urinary protein loss, and other consequences of advanced kidney disease. Although eGFR was included in the primary model and proteinuria was examined in sensitivity analysis, residual entanglement between nutritional risk and renal disease severity cannot be excluded. CONUT also does not assess muscle wasting, dietary intake, or energy balance, which are central to the protein-energy wasting construct. Third, repeated tongue-image acquisitions and independent duplicate measurements were not available; therefore, intra-rater or inter-rater ICCs and coefficients of variation could not be estimated. Standardised acquisition and automated feature extraction may reduce operator-dependent variation, but they do not replace formal technical repeatability assessment. Fourth, the study was conducted at two tertiary referral centres in Beijing, limiting generalisability to other care settings and populations. Fifth, although the overall sample included 392 patients, precision remained limited for the modest tongue-feature effect sizes and borderline associations observed. The severe CONUT category included only 11 patients, precluding reliable severity-specific regression, and the exploratory eGFR- and sex-stratified estimates had wide confidence intervals. Raw sex interaction signals did not remain significant after BH correction and require independent confirmation. Sixth, the tongue-feature effect sizes were modest, and no screening association survived FDR correction across all 51 features; tongue features should therefore not be interpreted as strong predictors in isolation. Finally, the pooled ordinal association for edge tongue saturation remained below conventional significance (OR = 1.29, *p* = 0.086), and the proportional odds assumption was not formally tested across the multiply imputed datasets. All tongue–CONUT findings should therefore be considered hypothesis-generating.

## 5 Conclusion

This study provides systematic exploratory evidence linking objective tongue phenotyping with CONUT-defined nutritional risk in patients with DKD. Two tongue features—middle tongue brightness and edge tongue saturation—retained modest, borderline associations with CONUT in the primary joint model. The coefficient for middle tongue brightness was comparatively stable across renal and glycaemic adjustment models, whereas the edge tongue saturation coefficient increased after adjustment for eGFR, consistent with statistical suppression. However, neither feature reached the conventional *p* < 0.05 threshold in the primary model, the sensitivity and ordinal estimates remained imprecise, and no screening association survived FDR correction across all 51 tongue features. These results should therefore be considered hypothesis-generating rather than evidence supporting immediate clinical screening. Future prospective studies with technical repeatability assessment, larger and more diverse samples, longitudinal nutritional measurements, and comprehensive nutritional criteria are warranted to determine whether objective tongue features provide incremental value for nutritional surveillance in DKD.

## Data Availability

The raw data supporting the conclusions of this article will be made available by the authors, without undue reservation.
