# Supplementary material for: Objective tongue phenotyping and nutritional risk in diabetic kidney disease: a dual-centre study linking quantified tongue features to the controlling nutritional status score
Source: Front Nutr. 2026 Aug 14;13:1902694. doi: 10.3389/fnut.2026.1902694 (PMC13521857; doi:10.3389/fnut.2026.1902694)
Supplement: Supplementary file 1 [file Data_Sheet_1.docx]

Supplementary Material

# 1 Supplementary Tables and Figures

## 1.1 Supplementary Tables

**Supplementary Table S1** Baseline clinical characteristics across four CONUT nutritional categories.

| **Variable** | **Normal (0-1)** N = 88 | **Light (2-4)** N = 196 | **Moderate (5-8)** N = 97 | **Severe (9-12)** N = 11 | **p-value** |
| --- | --- | --- | --- | --- | --- |
| **Age** | 58 (47, 68) | 63 (56, 71) | 61 (55, 68) | 66 (54, 74) | 0.006 |
| **Sex** |  |  |  |  | 0.2 |
| female | 35 (40%) | 59 (30%) | 27 (28%) | 2 (18%) |  |
| male | 53 (60%) | 137 (70%) | 70 (72%) | 9 (82%) |  |
| **Study centre** |  |  |  |  | 0.020 |
| center1 | 80 (91%) | 173 (88%) | 78 (80%) | 7 (64%) |  |
| center2 | 8 (9.1%) | 23 (12%) | 19 (20%) | 4 (36%) |  |
| **eGFR (CKD-EPI)** | 71 (40, 97) | 47 (19, 74) | 16 (9, 39) | 16 (10, 20) | <0.001 |
| **Serum creatinine (SCr)** | 98 (76, 159) | 138 (90, 281) | 311 (167, 567) | 378 (211, 531) | <0.001 |
| **Blood urea nitrogen (BUN)** | 9 (7, 13) | 11 (7, 16) | 16 (11, 24) | 21 (12, 27) | <0.001 |
| **Albumin (ALB)** | 41.3 (37.9, 43.8) | 37.8 (34.6, 41.7) | 31.5 (28.0, 34.4) | 25.6 (20.6, 28.5) | <0.001 |
| **Lymphocyte count (LY#)** | 1.96 (1.68, 2.23) | 1.50 (1.20, 1.84) | 1.03 (0.80, 1.42) | 0.92 (0.58, 1.10) | <0.001 |
| **Total cholesterol (TC)** | 5.44 (4.84, 6.08) | 4.02 (3.45, 5.01) | 3.52 (2.85, 4.72) | 3.65 (2.55, 3.71) | <0.001 |
| **Fasting glucose (Glu)** | 8.6 (6.3, 11.2) | 7.3 (6.1, 9.6) | 6.9 (5.4, 9.9) | 5.4 (5.1, 7.1) | 0.003 |
| **Glycated haemoglobin (HbA1c)** | 8.50 (7.27, 9.79) | 7.18 (6.33, 8.20) | 7.00 (6.10, 7.60) | 6.60 (5.80, 8.10) | <0.001 |
| **C-reactive protein (CRP)** | 1 (1, 3) | 1 (1, 3) | 1 (1, 10) | 2 (1, 36) | 0.13 |
| **White blood cell count (WBC)** | 6.72 (5.97, 7.77) | 6.60 (5.58, 8.10) | 5.80 (4.60, 7.20) | 6.14 (5.20, 8.39) | <0.001 |
| **Haemoglobin (HGB)** | 133 (122, 150) | 118 (102, 137) | 104 (90, 114) | 94 (79, 126) | <0.001 |
| **Platelet count (PLT)** | 229 (193, 264) | 208 (175, 270) | 195 (140, 251) | 169 (120, 219) | <0.001 |
| **Triglycerides (TG)** | 2.22 (1.40, 3.10) | 1.73 (1.18, 2.46) | 1.50 (0.98, 2.15) | 1.21 (0.83, 1.98) | <0.001 |
| **Low-density lipoprotein cholesterol (LDL-C)** | 3.15 (2.66, 3.81) | 2.32 (1.86, 2.92) | 1.98 (1.46, 2.59) | 1.82 (1.34, 2.52) | <0.001 |
| **High-density lipoprotein cholesterol (HDL-C)** | 1.11 (0.97, 1.33) | 1.06 (0.91, 1.29) | 1.03 (0.86, 1.20) | 1.00 (0.88, 1.21) | 0.093 |
| **Potassium (K)** | 4.32 (3.91, 4.55) | 4.44 (4.05, 4.79) | 4.56 (3.95, 5.04) | 4.23 (4.09, 4.50) | 0.048 |
| **Calcium (Ca)** | 2.34 (2.26, 2.44) | 2.26 (2.14, 2.35) | 2.06 (1.93, 2.19) | 1.90 (1.83, 2.06) | <0.001 |
| **Phosphorus (P)** | 1.30 (1.15, 1.43) | 1.32 (1.16, 1.47) | 1.40 (1.17, 1.78) | 1.50 (1.13, 1.68) | 0.008 |
| **Carbon dioxide combining power (CO₂CP)** | 25.5 (23.9, 27.3) | 24.6 (22.5, 27.2) | 24.2 (21.8, 26.0) | 22.5 (20.3, 25.8) | 0.001 |
| **24-hour urinary protein** | 641 (327, 2,430) | 1,931 (324, 4,728) | 4,390 (1,935, 8,017) | 5,860 (1,631, 10,786) | <0.001 |
| **Urine protein (dipstick)** |  |  |  |  | <0.001 |
| 0 | 26 (30%) | 51 (26%) | 9 (9.3%) | 0 (0%) |  |
| 1 | 11 (13%) | 21 (11%) | 1 (1.0%) | 0 (0%) |  |
| 2 | 15 (17%) | 22 (11%) | 6 (6.2%) | 0 (0%) |  |
| 3 | 21 (24%) | 45 (23%) | 28 (29%) | 4 (36%) |  |
| 4 | 15 (17%) | 57 (29%) | 52 (54%) | 7 (64%) |  |
| 5 | 0 (0%) | 0 (0%) | 1 (1.0%) | 0 (0%) |  |
| **Urine occult blood (dipstick)** |  |  |  |  | 0.031 |
| 0 | 57 (65%) | 124 (63%) | 54 (56%) | 4 (36%) |  |
| 1 | 13 (15%) | 20 (10%) | 16 (16%) | 0 (0%) |  |
| 2 | 12 (14%) | 22 (11%) | 9 (9.3%) | 4 (36%) |  |
| 3 | 2 (2.3%) | 23 (12%) | 13 (13%) | 3 (27%) |  |
| 4 | 4 (4.5%) | 7 (3.6%) | 5 (5.2%) | 0 (0%) |  |
| 5 | 0 (0%) | 0 (0%) | 0 (0%) | 0 (0%) |  |
| **Urine glucose (dipstick)** |  |  |  |  | 0.5 |
| 0 | 19 (22%) | 60 (31%) | 24 (25%) | 5 (45%) |  |
| 1 | 9 (10%) | 18 (9.2%) | 13 (13%) | 2 (18%) |  |
| 2 | 14 (16%) | 26 (13%) | 15 (15%) | 1 (9.1%) |  |
| 3 | 15 (17%) | 43 (22%) | 26 (27%) | 2 (18%) |  |
| 4 | 25 (28%) | 41 (21%) | 17 (18%) | 1 (9.1%) |  |
| 5 | 6 (6.8%) | 8 (4.1%) | 2 (2.1%) | 0 (0%) |  |
| **Urine ketones (dipstick)** |  |  |  |  | 0.2 |
| 0 | 77 (88%) | 186 (95%) | 93 (96%) | 10 (91%) |  |
| 1 | 7 (8.0%) | 5 (2.6%) | 3 (3.1%) | 0 (0%) |  |
| 2 | 3 (3.4%) | 5 (2.6%) | 1 (1.0%) | 1 (9.1%) |  |
| 3 | 1 (1.1%) | 0 (0%) | 0 (0%) | 0 (0%) |  |
| 4 | 0 (0%) | 0 (0%) | 0 (0%) | 0 (0%) |  |
| 5 | 0 (0%) | 0 (0%) | 0 (0%) | 0 (0%) |  |
| **Albumin subscore (ALB score)** |  |  |  |  | <0.001 |
| 0 | 88 (100%) | 142 (72%) | 19 (20%) | 0 (0%) |  |
| 2 | 0 (0%) | 46 (23%) | 42 (43%) | 0 (0%) |  |
| 4 | 0 (0%) | 8 (4.1%) | 24 (25%) | 6 (55%) |  |
| 6 | 0 (0%) | 0 (0%) | 12 (12%) | 5 (45%) |  |
| **Lymphocyte subscore (LY# score)** |  |  |  |  | <0.001 |
| 0 | 71 (81%) | 85 (43%) | 13 (13%) | 0 (0%) |  |
| 1 | 17 (19%) | 72 (37%) | 25 (26%) | 2 (18%) |  |
| 2 | 0 (0%) | 31 (16%) | 36 (37%) | 6 (55%) |  |
| 3 | 0 (0%) | 8 (4.1%) | 23 (24%) | 3 (27%) |  |
| **Cholesterol subscore (TC score)** |  |  |  |  | <0.001 |
| 0 | 53 (60%) | 42 (21%) | 17 (18%) | 1 (9.1%) |  |
| 1 | 35 (40%) | 49 (25%) | 17 (18%) | 1 (9.1%) |  |
| 2 | 0 (0%) | 76 (39%) | 29 (30%) | 5 (45%) |  |
| 3 | 0 (0%) | 29 (15%) | 34 (35%) | 4 (36%) |  |

Data are presented as median (interquartile range) for continuous variables, and as frequency (percentage) for categorical variables. CONUT nutritional categories: normal, 0–1; light malnutrition, 2–4; moderate malnutrition, 5–8; severe malnutrition, 9–12. Values are derived from the first imputed dataset. Between-group comparisons used the Kruskal–Wallis test for continuous variables and the Pearson chi-square test for categorical variables. Urine dipstick grades are coded as: 0 = negative; 1 = trace (±); 2 = 1+; 3 = 2+; 4 = 3+; 5 = 4+. CONUT albumin subscore: 0 = ALB ≥35 g/L; 2 = 30–34.9 g/L; 4 = 25–29.9 g/L; 6 = <25 g/L. CONUT lymphocyte subscore: 0 = LY# ≥1.6 ×10⁹/L; 1 = 1.2–1.59; 2 = 0.8–1.19; 3 = <0.8 ×10⁹/L. CONUT cholesterol subscore: 0 = TC ≥5.18 mmol/L; 1 = 4.14–5.17; 2 = 3.11–4.13; 3 = <3.11 mmol/L. CONUT, Controlling Nutritional Status score; eGFR, estimated glomerular filtration rate (CKD-EPI equation).

**Supplementary Table S2** Tongue features across four CONUT nutritional categories.

| **Tongue feature** | **Normal (0-1)** N = 88 | **Light (2-4)** N = 196 | **Moderate (5-8)** N = 97 | **Severe (9-12)** N = 11 | **p-value** |
| --- | --- | --- | --- | --- | --- |
| **Coating ratio (%)** | 29 (18, 41) | 31 (21, 41) | 34 (21, 42) | 34 (20, 41) | 0.5 |
| **Tip tongue colour, H (hue)** | 172.7 (171.2, 174.2) | 172.3 (170.1, 174.1) | 172.8 (171.1, 174.3) | 171.1 (167.0, 172.6) | 0.2 |
| **Tip tongue colour, S (saturation)** | 76 (67, 92) | 71 (64, 87) | 73 (66, 88) | 78 (68, 101) | 0.10 |
| **Tip tongue colour, V (brightness)** | 189 (181, 195) | 188 (177, 194) | 185 (171, 192) | 186 (163, 189) | 0.042 |
| **Tip coating colour, H** | 104 (16, 158) | 121 (17, 160) | 98 (9, 156) | 32 (2, 163) | 0.3 |
| **Tip coating colour, S** | 56 (35, 71) | 51 (35, 73) | 59 (35, 75) | 70 (31, 76) | 0.6 |
| **Tip coating colour, V** | 169 (110, 212) | 180 (133, 219) | 162 (117, 208) | 163 (123, 214) | 0.050 |
| **Middle tongue colour, H** | 173.2 (170.4, 175.9) | 173.1 (170.1, 175.9) | 173.5 (170.6, 176.0) | 171.0 (165.5, 173.4) | 0.3 |
| **Middle tongue colour, S** | 54 (47, 63) | 55 (45, 65) | 60 (51, 67) | 65 (53, 76) | 0.062 |
| **Middle tongue colour, V** | 196 (186, 199) | 196 (187, 199) | 193 (183, 198) | 188 (173, 196) | 0.028 |
| **Middle coating colour, H** | 145 (11, 166) | 156 (14, 169) | 125 (7, 165) | 36 (4, 165) | 0.069 |
| **Middle coating colour, S** | 34 (31, 48) | 33 (29, 46) | 34 (29, 58) | 33 (28, 67) | 0.3 |
| **Middle coating colour, V** | 213 (184, 221) | 215 (190, 223) | 207 (182, 218) | 195 (170, 219) | 0.028 |
| **Root tongue colour, H** | 170 (156, 175) | 170 (153, 174) | 170 (148, 174) | 171 (162, 174) | >0.9 |
| **Root tongue colour, S** | 61 (52, 70) | 59 (49, 74) | 60 (51, 76) | 59 (49, 107) | 0.8 |
| **Root tongue colour, V** | 165 (140, 181) | 166 (140, 184) | 154 (131, 178) | 155 (136, 177) | 0.2 |
| **Root coating colour, H** | 22 (5, 145) | 23 (6, 158) | 19 (5, 128) | 44 (8, 162) | 0.5 |
| **Root coating colour, S** | 57 (36, 71) | 51 (31, 68) | 57 (34, 75) | 37 (31, 88) | 0.2 |
| **Root coating colour, V** | 162 (140, 191) | 173 (145, 194) | 163 (140, 186) | 155 (125, 193) | 0.3 |
| **Edge tongue colour, H** | 173 (170, 175) | 173 (171, 174) | 173 (169, 175) | 170 (160, 174) | 0.2 |
| **Edge tongue colour, S** | 67 (60, 76) | 67 (58, 83) | 74 (61, 98) | 91 (65, 102) | 0.038 |
| **Edge tongue colour, V** | 179 (160, 188) | 183 (162, 189) | 168 (148, 186) | 162 (138, 181) | 0.001 |
| **Edge coating colour, H** | 111 (14, 152) | 122 (58, 158) | 102 (16, 158) | 144 (3, 158) | 0.6 |
| **Edge coating colour, S** | 61 (41, 85) | 57 (35, 78) | 65 (38, 87) | 78 (34, 122) | 0.3 |
| **Edge coating colour, V** | 149 (113, 200) | 168 (117, 201) | 151 (107, 194) | 147 (90, 194) | 0.2 |
| **Whole-tongue colour, H** | 160 (141, 168) | 163 (134, 169) | 157 (117, 168) | 166 (109, 171) | 0.3 |
| **Whole-tongue colour, S** | 55 (48, 63) | 53 (43, 65) | 58 (45, 69) | 63 (46, 83) | 0.10 |
| **Whole-tongue colour, V** | 202 (178, 210) | 204 (180, 212) | 196 (172, 209) | 180 (165, 205) | 0.020 |
| **Coating 1st-order colour moment, B channel** | 113 (91, 125) | 116 (96, 131) | 99 (87, 123) | 98 (73, 124) | 0.005 |
| **Coating 1st-order colour moment, G channel** | 105 (88, 119) | 108 (91, 123) | 96 (84, 114) | 89 (71, 115) | 0.004 |
| **Coating 1st-order colour moment, R channel** | 133 (115, 147) | 137 (119, 149) | 124 (111, 139) | 122 (104, 137) | 0.003 |
| **Coating 2nd-order colour moment, B channel** | 80 (67, 87) | 83 (71, 89) | 80 (65, 88) | 69 (58, 90) | 0.2 |
| **Coating 2nd-order colour moment, G channel** | 76 (66, 83) | 77 (67, 85) | 75 (63, 83) | 63 (58, 85) | 0.094 |
| **Coating 2nd-order colour moment, R channel** | 92 (83, 100) | 95 (86, 101) | 92 (82, 100) | 88 (79, 99) | 0.3 |
| **Coating 3rd-order colour moment, B channel** | -65 (-74, -50) | -66 (-78, -52) | -53 (-72, -44) | -57 (-71, -31) | 0.009 |
| **Coating 3rd-order colour moment, G channel** | -60 (-70, -49) | -62 (-72, -50) | -55 (-67, -42) | -52 (-67, -23) | 0.008 |
| **Coating 3rd-order colour moment, R channel** | -79 (-89, -67) | -81 (-90, -69) | -74 (-84, -61) | -74 (-83, -48) | 0.006 |
| **Coating 1st-order colour moment, BGR composite** | 117 (99, 130) | 121 (102, 133) | 106 (95, 126) | 102 (84, 127) | 0.004 |
| **Coating 2nd-order colour moment, BGR composite** | 84 (74, 91) | 86 (76, 92) | 85 (72, 91) | 76 (67, 93) | 0.2 |
| **Coating 3rd-order colour moment, BGR composite** | -66 (-76, -52) | -68 (-79, -53) | -57 (-73, -41) | -57 (-73, -19) | 0.006 |
| **Coating texture: correlation (COR)** | 0.951 (0.934, 0.957) | 0.952 (0.936, 0.957) | 0.952 (0.942, 0.957) | 0.943 (0.922, 0.958) | 0.8 |
| **Coating texture: contrast (CON)** | 752 (633, 879) | 781 (618, 925) | 710 (520, 830) | 760 (421, 837) | 0.022 |
| **Coating texture: energy** | 0.59 (0.53, 0.64) | 0.58 (0.51, 0.65) | 0.59 (0.50, 0.66) | 0.55 (0.45, 0.64) | 0.6 |
| **Coating texture: angular second moment (ASM)** | 0.34 (0.28, 0.41) | 0.34 (0.26, 0.42) | 0.34 (0.25, 0.43) | 0.30 (0.21, 0.41) | 0.6 |
| **Coating texture: inverse difference moment (IDM)** | 7.10 (6.10, 8.35) | 7.06 (5.98, 8.24) | 6.69 (5.74, 8.13) | 8.18 (5.66, 9.39) | 0.5 |
| **Coating texture: entropy** | 3.34 (3.01, 3.81) | 3.37 (2.97, 3.86) | 3.40 (2.88, 3.90) | 3.64 (3.04, 4.26) | 0.5 |
| **Coating texture: roughness** | 6.35 (5.69, 7.19) | 6.49 (5.71, 7.35) | 6.33 (5.56, 7.32) | 6.95 (6.37, 7.83) | 0.4 |
| **Coating texture: grey mean** | 175 (146, 191) | 180 (153, 196) | 167 (145, 190) | 146 (128, 189) | 0.035 |
| **Coating thickness grade (0–3)** |  |  |  |  | 0.8 |
| 0 | 7 (8.0%) | 12 (6.1%) | 8 (8.2%) | 1 (9.1%) |  |
| 1 | 45 (51%) | 85 (43%) | 39 (40%) | 7 (64%) |  |
| 2 | 18 (20%) | 51 (26%) | 25 (26%) | 1 (9.1%) |  |
| 3 | 18 (20%) | 48 (24%) | 25 (26%) | 2 (18%) |  |
| **Tooth marks: present, n (%)** | 60 (68%) | 133 (68%) | 59 (61%) | 8 (73%) | 0.6 |
| **Fissures: present, n (%)** | 21 (24%) | 54 (28%) | 21 (22%) | 2 (18%) | 0.7 |

Data are presented as median (interquartile range) for continuous tongue features, and as frequency (percentage) for ordinal and binary features. CONUT nutritional categories: normal, 0–1; light malnutrition, 2–4; moderate malnutrition, 5–8; severe malnutrition, 9–12. Values are derived from the first imputed dataset. Between-group comparisons used the Kruskal–Wallis test for continuous features and the Pearson chi-square test for categorical features. No tongue features were missing. HSV colour model: H = hue (0–180); S = saturation (0–255); V = brightness/value (0–255). RGB colour model: R = red, G = green, B = blue (0–255). Colour moments and GLCM descriptors were computed from the tongue coating region. Coating thickness grade: 0 = absent; 1 = mild; 2 = moderate; 3 = severe. Tooth marks and fissures are reported as the number (percentage) of patients with the feature present.

ASM, angular second moment; BGR, blue-green-red composite; CONUT, Controlling Nutritional Status score; COR, correlation; CON, contrast; GLCM, grey-level co-occurrence matrix; H, hue; IDM, inverse difference moment; IQR, interquartile range; RGB, red-green-blue; S, saturation; V, value (brightness).

**Supplementary Table S3** Rubin-pooled partial Spearman correlations between all 51 tongue features and CONUT score.

| **Tongue feature** | **Variable type** | **Pooled r** | **95% CI** | **P value** | **P (FDR-adj)** |
| --- | --- | --- | --- | --- | --- |
| Edge tongue colour, S (saturation) | continuous | 0.121 | 0.018 to 0.222 | 0.021 | 0.363 |
| Edge tongue colour, V (brightness) | continuous | -0.105 | -0.205 to -0.003 | 0.044 | 0.363 |
| Coating 3rd-order colour moment, G channel | continuous | 0.104 | 0.002 to 0.203 | 0.045 | 0.363 |
| Coating 3rd-order colour moment, BGR composite | continuous | 0.102 | 0.001 to 0.202 | 0.049 | 0.363 |
| Coating 1st-order colour moment, G channel | continuous | -0.102 | -0.201 to -0.000 | 0.049 | 0.363 |
| Coating 3rd-order colour moment, R channel | continuous | 0.101 | -0.001 to 0.200 | 0.053 | 0.363 |
| Coating 1st-order colour moment, R channel | continuous | -0.099 | -0.199 to 0.003 | 0.056 | 0.363 |
| Coating 1st-order colour moment, BGR composite | continuous | -0.098 | -0.198 to 0.003 | 0.057 | 0.363 |
| Coating 3rd-order colour moment, B channel | continuous | 0.093 | -0.008 to 0.193 | 0.071 | 0.383 |
| Coating 1st-order colour moment, B channel | continuous | -0.091 | -0.191 to 0.010 | 0.077 | 0.383 |
| Middle tongue colour, V | continuous | -0.089 | -0.188 to 0.012 | 0.083 | 0.383 |
| Edge tongue colour, H (hue) | continuous | -0.077 | -0.178 to 0.026 | 0.144 | 0.613 |
| Tip tongue colour, V (brightness) | continuous | -0.072 | -0.173 to 0.031 | 0.171 | 0.671 |
| Middle tongue colour, H | continuous | -0.066 | -0.166 to 0.036 | 0.203 | 0.740 |
| Coating texture: contrast (CON) | continuous | -0.063 | -0.163 to 0.039 | 0.227 | 0.771 |
| Tip tongue colour, H (hue) | continuous | -0.058 | -0.159 to 0.044 | 0.266 | 0.825 |
| Tip coating colour, V | continuous | -0.054 | -0.155 to 0.049 | 0.302 | 0.825 |
| Whole-tongue colour, S | continuous | 0.053 | -0.049 to 0.154 | 0.310 | 0.825 |
| Edge coating colour, V | continuous | -0.052 | -0.151 to 0.049 | 0.316 | 0.825 |
| Middle coating colour, V | continuous | -0.051 | -0.152 to 0.051 | 0.323 | 0.825 |
| Whole-tongue colour, H | continuous | 0.048 | -0.054 to 0.149 | 0.358 | 0.831 |
| Whole-tongue colour, V | continuous | -0.046 | -0.146 to 0.055 | 0.372 | 0.831 |
| Tip tongue colour, S (saturation) | continuous | 0.045 | -0.058 to 0.146 | 0.394 | 0.831 |
| Root coating colour, H | continuous | 0.043 | -0.060 to 0.144 | 0.413 | 0.831 |
| Coating ratio (%) | continuous | -0.042 | -0.144 to 0.061 | 0.427 | 0.831 |
| Root tongue colour, V | continuous | -0.040 | -0.140 to 0.061 | 0.439 | 0.831 |
| Edge coating colour, H | continuous | 0.039 | -0.062 to 0.139 | 0.452 | 0.831 |
| Tooth marks: present, n (%) | binary | -0.038 | -0.139 to 0.063 | 0.459 | 0.831 |
| Middle tongue colour, S | continuous | 0.036 | -0.065 to 0.137 | 0.484 | 0.831 |
| Coating texture: grey mean | continuous | -0.036 | -0.136 to 0.066 | 0.490 | 0.831 |
| Fissures: present, n (%) | binary | -0.035 | -0.137 to 0.068 | 0.505 | 0.831 |
| Edge coating colour, S | continuous | 0.031 | -0.070 to 0.132 | 0.546 | 0.870 |
| Tip coating colour, H | continuous | -0.030 | -0.132 to 0.073 | 0.567 | 0.877 |
| Coating 2nd-order colour moment, G channel | continuous | -0.028 | -0.129 to 0.074 | 0.593 | 0.890 |
| Root coating colour, S | continuous | -0.026 | -0.127 to 0.076 | 0.621 | 0.904 |
| Coating texture: correlation (COR) | continuous | -0.024 | -0.126 to 0.078 | 0.642 | 0.909 |
| Coating texture: roughness | continuous | 0.019 | -0.082 to 0.120 | 0.711 | 0.965 |
| Coating thickness grade (0–3) | ordinal | 0.019 | -0.084 to 0.121 | 0.719 | 0.965 |
| Coating texture: inverse difference moment (IDM) | continuous | -0.016 | -0.118 to 0.085 | 0.752 | 0.979 |
| Root tongue colour, H | continuous | 0.015 | -0.087 to 0.117 | 0.770 | 0.979 |
| Coating 2nd-order colour moment, B channel | continuous | -0.012 | -0.113 to 0.089 | 0.817 | 0.979 |
| Coating 2nd-order colour moment, BGR composite | continuous | -0.012 | -0.113 to 0.089 | 0.821 | 0.979 |
| Root tongue colour, S | continuous | -0.011 | -0.113 to 0.090 | 0.825 | 0.979 |
| Root coating colour, V | continuous | -0.004 | -0.107 to 0.099 | 0.935 | 0.998 |
| Coating texture: energy | continuous | -0.003 | -0.104 to 0.098 | 0.948 | 0.998 |
| Coating texture: angular second moment (ASM) | continuous | -0.003 | -0.104 to 0.098 | 0.949 | 0.998 |
| Middle coating colour, S | continuous | -0.002 | -0.103 to 0.099 | 0.969 | 0.998 |
| Middle coating colour, H | continuous | 0.001 | -0.102 to 0.104 | 0.984 | 0.998 |
| Coating texture: entropy | continuous | 0.001 | -0.100 to 0.102 | 0.984 | 0.998 |
| Coating 2nd-order colour moment, R channel | continuous | -0.001 | -0.102 to 0.100 | 0.987 | 0.998 |
| Tip coating colour, S | continuous | 0.000 | -0.102 to 0.101 | 0.998 | 0.998 |

Partial Spearman correlations were estimated via rank-based residualisation adjusted for age, sex, study centre, eGFR, and HbA1c, pooled across 20 imputed datasets using Fisher z-transformation and Rubin's rules (variance approximation: 1/(n − k − 3)). The Benjamini–Hochberg (BH) false discovery rate (FDR) procedure was applied across all 51 features simultaneously. Features are ordered by unadjusted pooled p-value (ascending). No feature survived FDR correction at q < 0.05; 11 features had pooled p < 0.10 and were retained as candidates for subsequent regression. The "Variable type" column indicates whether the feature is continuous, ordinal, or binary; all three types were analysed using the same rank-based residualisation approach.

BH, Benjamini–Hochberg; CI, confidence interval; CONUT, Controlling Nutritional Status score; eGFR, estimated glomerular filtration rate; FDR, false discovery rate; HbA1c, glycated haemoglobin; HSV, hue-saturation-value; P (FDR-adj), Benjamini–Hochberg FDR-adjusted p-value; r, partial Spearman correlation coefficient.

**Supplementary Table S4** Variance inflation factors for the primary multivariable model.

| **Model term** | **VIF** |
| --- | --- |
| Coating 1st-order colour moment, G [z-scored] | 4.008 |
| Edge tongue brightness (V) [z-scored] | 3.316 |
| Coating 3rd-order colour moment, G [z-scored] | 2.763 |
| Edge tongue saturation (S) [z-scored] | 1.973 |
| Middle tongue brightness (V) [z-scored] | 1.681 |
| Centre 2 vs 1 | 1.461 |
| eGFR | 1.186 |
| HbA1c | 1.175 |
| Sex: male | 1.108 |
| Age (years) | 1.090 |

VIFs were computed from the first imputed dataset for the primary multivariable model, which included five tongue features (z-score standardised) and five covariates (age, sex, study centre, eGFR, and HbA1c). All VIFs were below 5.0 (range 1.09–4.01), indicating acceptable multicollinearity throughout. The elevated VIF for the coating 1st-order colour moment (G channel; VIF = 4.01) reflects partial correlation with edge tongue brightness and saturation features, which share an underlying dimension of tongue colour change; this level of collinearity does not invalidate the model estimates.

eGFR, estimated glomerular filtration rate; HbA1c, glycated haemoglobin; VIF, variance inflation factor.

**Supplementary Table S5** Rubin-pooled ordinal logistic regression of tongue features against CONUT nutritional category.

| **Tongue feature** | **log-OR** | **OR** | **95% CI (OR)** | **P value** | **P (FDR-adj)** |
| --- | --- | --- | --- | --- | --- |
| Edge tongue saturation (S) | 0.251 | 1.285 | 0.965 to 1.712 | 0.086 | 0.430 |
| Middle tongue brightness (V) | -0.155 | 0.856 | 0.669 to 1.095 | 0.216 | 0.541 |
| Coating 1st-order colour moment, G channel | -0.088 | 0.915 | 0.615 to 1.363 | 0.665 | 0.981 |
| Edge tongue brightness (V) | -0.010 | 0.990 | 0.688 to 1.426 | 0.958 | 0.981 |
| Coating 3rd-order colour moment, G channel | 0.004 | 1.004 | 0.717 to 1.406 | 0.981 | 0.981 |

Odds ratios (ORs) represent the odds of being in a higher CONUT nutritional category (normal < light < moderate < severe) per one standard deviation increase in each tongue feature, estimated using the proportional odds model (R function MASS::polr). Log-ORs were estimated within each of 20 imputed datasets and pooled on the log-odds scale using Rubin's rules; pooled log-ORs were exponentiated to yield ORs with 95% confidence intervals. The model was adjusted for age, sex, study centre, eGFR, and HbA1c. Features are ordered by pooled p-value (ascending). No feature survived Benjamini–Hochberg FDR correction at q < 0.05. Results are presented as a supplementary exploratory analysis to complement the primary continuous CONUT regression.

CI, confidence interval; CONUT, Controlling Nutritional Status score; eGFR, estimated glomerular filtration rate; FDR, false discovery rate; HbA1c, glycated haemoglobin; log-OR, natural logarithm of the odds ratio; OR, odds ratio; P (FDR-adj), Benjamini–Hochberg FDR-adjusted p-value; SD, standard deviation.

**Supplementary Table S6** Full multivariable model coefficients including covariates.

| **Term** | **Type** | **β** | **SE** | **95% CI** | **P value** |
| --- | --- | --- | --- | --- | --- |
| eGFR (per mL/min/1.73 m²) | Covariate | -0.029 | 0.003 | -0.035 to -0.022 | <0.001 |
| Sex: male vs female | Covariate | 0.692 | 0.234 | 0.232 to 1.151 | 0.003 |
| Age (per year) | Covariate | 0.021 | 0.009 | 0.004 to 0.039 | 0.015 |
| HbA1c (per 1%) | Covariate | -0.104 | 0.057 | -0.216 to 0.008 | 0.067 |
| Centre 2 vs centre 1 | Covariate | 0.194 | 0.367 | -0.527 to 0.914 | 0.598 |
| Edge tongue saturation (S) [per SD] | Tongue feature | 0.269 | 0.149 | -0.023 to 0.560 | 0.071 |
| Middle tongue brightness (V) [per SD] | Tongue feature | -0.234 | 0.133 | -0.495 to 0.026 | 0.078 |
| Coating 1st-order moment, G channel [per SD] | Tongue feature | -0.087 | 0.208 | -0.494 to 0.321 | 0.677 |
| Coating 3rd-order moment, G channel [per SD] | Tongue feature | 0.069 | 0.172 | -0.269 to 0.406 | 0.689 |
| Edge tongue brightness (V) [per SD] | Tongue feature | 0.067 | 0.190 | -0.305 to 0.439 | 0.724 |

Complete coefficient table for the primary multivariable linear regression model, including both tongue features (z-score standardised) and all covariates. β coefficients represent the change in CONUT score per one standard deviation increase for tongue features, and per one unit increase for continuous covariates (age: per year; eGFR: per mL/min/1.73 m²; HbA1c: per 1%). All estimates are Rubin-pooled across 20 imputed datasets. The complete model is reproduced here to facilitate detailed review of all covariate and tongue-feature estimates. Features are ordered by type (covariates first, by ascending p-value), then by tongue features (ascending p-value).

CI, confidence interval; CONUT, Controlling Nutritional Status score; eGFR, estimated glomerular filtration rate; HbA1c, glycated haemoglobin; SD, standard deviation; SE, standard error.

**Supplementary Table S7** eGFR-stratified Rubin-pooled joint multivariable models.

| **eGFR stratum** | **Tongue feature** | **β** | **SE** | **95% CI** | **P value** |
| --- | --- | --- | --- | --- | --- |
| eGFR ≥45 | Middle tongue brightness (V) | -0.345 | 0.158 | -0.657 to -0.034 | 0.030 |
| eGFR ≥45 | Coating 1st-order colour moment, G channel | 0.429 | 0.249 | -0.062 to 0.920 | 0.086 |
| eGFR ≥45 | Edge tongue brightness (V) | 0.082 | 0.235 | -0.381 to 0.546 | 0.727 |
| eGFR ≥45 | Edge tongue saturation (S) | 0.395 | 0.199 | 0.002 to 0.787 | 0.049 |
| eGFR ≥45 | Coating 3rd-order colour moment, G channel | 0.330 | 0.195 | -0.056 to 0.715 | 0.093 |
| eGFR <45 | Middle tongue brightness (V) | -0.229 | 0.206 | -0.634 to 0.177 | 0.268 |
| eGFR <45 | Coating 1st-order colour moment, G channel | -0.417 | 0.320 | -1.050 to 0.215 | 0.194 |
| eGFR <45 | Edge tongue brightness (V) | 0.216 | 0.287 | -0.350 to 0.782 | 0.452 |
| eGFR <45 | Edge tongue saturation (S) | 0.269 | 0.211 | -0.148 to 0.685 | 0.205 |
| eGFR <45 | Coating 3rd-order colour moment, G channel | -0.087 | 0.276 | -0.631 to 0.458 | 0.754 |

β coefficients represent the change in continuous CONUT score per one standard deviation increase in each tongue feature. Within each eGFR stratum, all five tongue features were entered simultaneously into a joint multivariable linear regression model adjusted for age, sex, study centre, continuous eGFR, and HbA1c. Continuous eGFR was retained in the models to account for residual variation within each stratum. Tongue features were z-score standardised using the mean and standard deviation of the first imputed dataset. Estimates were pooled across 20 imputed datasets using Rubin’s rules. Sample sizes ranged from 181 to 182 for eGFR ≥45 mL/min/1.73 m² and from 210 to 211 for eGFR <45 mL/min/1.73 m² across imputations. P values are unadjusted and should be interpreted as exploratory.

CI, confidence interval; CONUT, Controlling Nutritional Status score; eGFR, estimated glomerular filtration rate; HbA1c, glycated haemoglobin; SD, standard deviation; SE, standard error.

**Supplementary Table S8** Rubin-pooled interactions between tongue features and continuous eGFR.

| **Tongue feature** | **Interaction β** | **SE** | **95% CI** | **P value** | **P (BH-adjusted)** |
| --- | --- | --- | --- | --- | --- |
| Middle tongue brightness (V) | 0.017 | 0.034 | -0.050 to 0.084 | 0.611 | 0.816 |
| Coating 1st-order colour moment, G channel | 0.029 | 0.031 | -0.033 to 0.090 | 0.360 | 0.816 |
| Edge tongue brightness (V) | -0.001 | 0.031 | -0.061 to 0.059 | 0.973 | 0.973 |
| Edge tongue saturation (S) | 0.017 | 0.030 | -0.042 to 0.077 | 0.567 | 0.816 |
| Coating 3rd-order colour moment, G channel | -0.015 | 0.032 | -0.078 to 0.049 | 0.653 | 0.816 |

Each row reports the interaction coefficient from a separate full-sample multivariable linear regression model. Each model simultaneously included all five tongue features, age, sex, study centre, eGFR, HbA1c, and one prespecified tongue feature × eGFR interaction term. eGFR was centred at 45 mL/min/1.73 m² and scaled per 10 mL/min/1.73 m²; therefore, the interaction β represents the change in the tongue feature–CONUT association per 10 mL/min/1.73 m² increase in eGFR. The corresponding tongue-feature main effect represents its estimated association at eGFR = 45 mL/min/1.73 m². Estimates were pooled across 20 imputed datasets using Rubin’s rules. Benjamini–Hochberg adjustment was applied across the five eGFR interaction tests. The analysis included 392 participants.

BH, Benjamini–Hochberg; CI, confidence interval; CONUT, Controlling Nutritional Status score; eGFR, estimated glomerular filtration rate; HbA1c, glycated haemoglobin; SE, standard error.

**Supplementary Table S9** Sex-stratified Rubin-pooled joint multivariable models.

| **Sex** | **Tongue feature** | **β** | **SE** | **95% CI** | **P value** |
| --- | --- | --- | --- | --- | --- |
| Female | Middle tongue brightness (V) | 0.193 | 0.335 | -0.470 to 0.857 | 0.565 |
| Female | Coating 1st-order colour moment, G channel | -0.738 | 0.413 | -1.558 to 0.081 | 0.077 |
| Female | Edge tongue brightness (V) | 0.583 | 0.370 | -0.151 to 1.317 | 0.118 |
| Female | Edge tongue saturation (S) | 0.419 | 0.260 | -0.097 to 0.935 | 0.110 |
| Female | Coating 3rd-order colour moment, G channel | 0.063 | 0.334 | -0.598 to 0.724 | 0.851 |
| Male | Middle tongue brightness (V) | -0.312 | 0.143 | -0.594 to -0.030 | 0.030 |
| Male | Coating 1st-order colour moment, G channel | 0.135 | 0.240 | -0.337 to 0.607 | 0.574 |
| Male | Edge tongue brightness (V) | -0.223 | 0.221 | -0.659 to 0.212 | 0.314 |
| Male | Edge tongue saturation (S) | 0.148 | 0.180 | -0.206 to 0.501 | 0.412 |
| Male | Coating 3rd-order colour moment, G channel | 0.078 | 0.200 | -0.316 to 0.471 | 0.698 |

β coefficients represent the change in continuous CONUT score per one standard deviation increase in each tongue feature. Female- and male-specific estimates were obtained from separate joint multivariable linear regression models in which all five tongue features were entered simultaneously. Models were adjusted for age, study centre, eGFR, and HbA1c; sex was omitted because the models were stratified by sex. Tongue features were z-score standardised using the mean and standard deviation of the first imputed dataset. Estimates were pooled across 20 imputed datasets using Rubin’s rules. The analyses included 123 female and 269 male participants. P values are unadjusted and should be interpreted as exploratory.

CI, confidence interval; CONUT, Controlling Nutritional Status score; eGFR, estimated glomerular filtration rate; HbA1c, glycated haemoglobin; SD, standard deviation; SE, standard error.

**Supplementary Table S10** Rubin-pooled interactions between tongue features and sex.

| Tongue feature | Interaction β (male vs female) | SE | 95% CI | P value | P (BH-adjusted) |
| --- | --- | --- | --- | --- | --- |
| Middle tongue brightness (V) | -0.476 | 0.237 | -0.942 to -0.010 | 0.045 | 0.113 |
| Coating 1st-order colour moment, G channel | 0.105 | 0.223 | -0.334 to 0.544 | 0.639 | 0.716 |
| Edge tongue brightness (V) | -0.475 | 0.223 | -0.914 to -0.035 | 0.034 | 0.113 |
| Edge tongue saturation (S) | 0.080 | 0.220 | -0.352 to 0.513 | 0.716 | 0.716 |
| Coating 3rd-order colour moment, G channel | -0.233 | 0.227 | -0.680 to 0.214 | 0.307 | 0.511 |

Each row reports the interaction coefficient from a separate full-sample multivariable linear regression model. Each model simultaneously included all five tongue features, age, sex, study centre, eGFR, HbA1c, and one prespecified tongue feature × sex interaction term. Female participants constituted the reference group; therefore, the interaction β represents the difference in the tongue feature–CONUT association between male and female participants (male minus female), while the corresponding tongue-feature main effect represents its estimated association in female participants. Estimates were pooled across 20 imputed datasets using Rubin’s rules. Benjamini–Hochberg adjustment was applied across the five sex interaction tests. The analysis included 392 participants.

BH, Benjamini–Hochberg; CI, confidence interval; CONUT, Controlling Nutritional Status score; eGFR, estimated glomerular filtration rate; HbA1c, glycated haemoglobin; SE, standard error.

## 1.2 Supplementary Figures

**Supplementary Figure S1.** **Covariate coefficients from the primary multivariable model.** Forest plot of Rubin-pooled regression coefficients for the five covariates (age, sex, study centre, eGFR, and HbA1c) in the primary multivariable linear regression model, derived from Supplementary Table S6. Each point represents the pooled β coefficient with horizontal bars indicating 95% confidence intervals. Red points indicate nominal p < 0.05; blue points indicate p ≥ 0.05. eGFR was the strongest predictor of CONUT (β = −0.029 per mL/min/1.73 m², p < 10⁻¹⁶), followed by male sex (β = +0.692, p = 0.003) and age (β = +0.021 per year, p = 0.015). HbA1c showed a borderline inverse association (β = −0.104, p = 0.067). Centre 2 did not contribute independently (β = +0.194, p = 0.598).

CONUT, Controlling Nutritional Status score; eGFR, estimated glomerular filtration rate; HbA1c, glycated haemoglobin.

**Supplementary Figure S2–S4.** **Scatter plots of the top three tongue features against CONUT score (first imputed dataset).** Each panel shows the unadjusted bivariate relationship between one tongue feature (x-axis) and continuous CONUT score (y-axis) in the first imputed dataset (n = 392). The blue regression line with grey 95% confidence band is shown. CONUT scores are displayed as discrete integers reflecting the counting nature of the score. (S2) Edge tongue saturation (S); (S3) Edge tongue brightness (V); (S4) Coating 3rd-order colour moment, G channel. Note that these unadjusted scatter plots are for visual reference only; the primary analyses are covariate-adjusted (see Tables 2–4).

CONUT, Controlling Nutritional Status score; S, saturation; V, value (brightness).

**Supplementary Figure S5. Distributions of edge tongue saturation and middle tongue brightness across CONUT nutritional categories.** Violin plots, box plots, and individual observations are shown for the normal (n = 88), light (n = 196), moderate (n = 97), and severe (n = 11) categories in the first imputed dataset. The figure is descriptive and no additional inferential test was performed. CONUT, Controlling Nutritional Status score; S, saturation; V, value (brightness).
